# Carbonic Anhydrases: Role in pH Control and Cancer

**DOI:** 10.3390/metabo8010019

**Published:** 2018-02-28

**Authors:** Mam Y. Mboge, Brian P. Mahon, Robert McKenna, Susan C. Frost

**Affiliations:** University of Florida, College of Medicine, Department of Biochemistry and Molecular Biology, P.O. Box 100245, Gainesville, FL 32610, USA; brian.mahon@nih.gov (B.P.M.); rmckenna@ufl.edu (R.M.)

**Keywords:** tumors, pH, carbonic anhydrases, metalloenzymes, carbonic anhydrase IX, carbonic anhydrase XII, cancer therapeutics, metabolism, tumor microenvironment, drug discovery

## Abstract

The pH of the tumor microenvironment drives the metastatic phenotype and chemotherapeutic resistance of tumors. Understanding the mechanisms underlying this pH-dependent phenomenon will lead to improved drug delivery and allow the identification of new therapeutic targets. This includes an understanding of the role pH plays in primary tumor cells, and the regulatory factors that permit cancer cells to thrive. Over the last decade, carbonic anhydrases (CAs) have been shown to be important mediators of tumor cell pH by modulating the bicarbonate and proton concentrations for cell survival and proliferation. This has prompted an effort to inhibit specific CA isoforms, as an anti-cancer therapeutic strategy. Of the 12 active CA isoforms, two, CA IX and XII, have been considered anti-cancer targets. However, other CA isoforms also show similar activity and tissue distribution in cancers and have not been considered as therapeutic targets for cancer treatment. In this review, we consider all the CA isoforms and their possible role in tumors and their potential as targets for cancer therapy.

## 1. Introduction

Carbonic anhydrases (CAs) have been studied for over 90 years. Since their first discovery in 1933, CAs have been at the forefront of scientific discovery; from basic enzymology, to the application of structural biology and in silico approaches to study protein dynamics. In addition, CAs have been shown to be important in drug discovery and clinical medicine. CAs comprise a family of metalloenzymes that catalyze the reversible hydration of CO_2_, in the presence of water, to HCO_3_^−^ with the release of a proton a mechanism that was first inferred in 1933 [1]. CAs are grouped into six evolutionary distinct classes (α, β, γ, δ, ζ, and η), which implies convergent evolution of a biochemical reaction essential for life processes, encompassing both prokaryotic and eukaryotic organisms and viruses. These ubiquitous enzymes play important roles in ion transport, acid–base regulation, gas exchange, photosynthesis and CO_2_ fixation [2,3]. The α-class, which is the best characterized of the six classes, is found primarily in vertebrates. The β-class is present in higher plants and some prokaryotes, γ is expressed in higher plants and prokaryotes, and the δ and ζ have only been observed in diatoms [2,4]. The most recently identified CA family is the η-class, which was discovered in the malaria pathogen, *Plasmodium falciparum* [5]. 

The human CAs (α-class) share the same 3D tertiary structure, but differ in sequence (Table 1). Furthermore, CAs are expressed in specific tissues and cellular compartments that differ in pH and metabolic rate, properties that drive the contributions of catalyzed CO_2_ reactions in many physiological processes. In humans, 15 CA isoforms are encoded. Among these, only 12 coordinate a zinc in the active site making them catalytically active (CAs I–IV, CAs VA–VB, CAs VI–VII, CA IX, and CAs XII–XIV). Isoforms CA VIII, X, and XI are termed CA-related proteins (CA-RPs) as they lack the required metal ion within the active site [6,7]. While the α-CAs were initially purified from bovine erythrocytes, studies in human erythrocytes revealed three CA isoforms designated as A and B, which we now know are identical, and C (now named CA I and CA II, respectively) [8,9]. Amino acid sequences and X-ray crystallography studies for both isoforms were reported during the 1970s [6,7,10,11]. During the same decade, CA III, a sulfonamide-resistant CA isoform, was discovered and purified from rabbit skeletal muscle [12]. In 1979, the secreted CA VI isoform was isolated from the saliva of sheep and later characterized in humans [13,14]. CA IV, a membrane-associated isoform, was purified during the 1980s [15,16,17,18], while cytosolic CA VII isoform [19], the membrane-bound isoforms CA IX [20,21], CA XII [22], and CA XIV [23,24], and the mitochondrial isoforms CA VA and CA VB [25,26,27] were all discovered in the 1990s. During the early 2000s, CA XIII was purified and characterized [28], whereas the CA-RPs were shown to be cytosolic proteins [29].

The α-CAs are involved in many physiological processes including respiration, pH regulation, Na^+^ retention, calcification, bone resorption, signal transduction, electrolyte secretion, gluconeogenesis, ureagenesis, and lipogenesis. Due to their broad roles in metabolism, CAs have served as therapeutic targets for several diseases, including glaucoma and epileptic seizures, altitude sickness, and more recently in the treatment of obesity and pain [30,31,32]. In addition, two of the membrane-bound CAs have been shown to be important for tumorigenesis [3,33,34,35,36,37,38,39,40,41]. CAs have also gained industrial interest as biocatalysts for carbon sequestration of fuel-gas and CO_2_ gas exchange in artificial lungs [42,43,44,45,46,47]. All of these applications are possible because of the favorable properties of CAs such as “fast” enzyme kinetics, easy expression, high solubility, and intermediate heat resistance [4,48]. The kinetic properties of the CAs are listed in Table 2. 

Of the 15 CA isoforms expressed in humans, only CA IX and CA XII have been implicated in cancer. These enzymes are transmembrane proteins in which their extracellular domain contains the catalytic activity, positioning them in the regulation of the tumor microenvironment. CA IX is of particular interest because of its high expression in solid tumors while exhibiting low expression in normal tissues [3,48,49,50]. Yet, reducing activity of either CA IX/XII activity appears to affect the pH of the tumor microenvironment reducing tumor cell survival and proliferation [33,51]. Taken together, these characteristics make CA IX/XII attractive as anti-cancer targets. Other isoforms may have targeting potential with respect to cancer, but little is known about their specific function even though there is evidence of their expression and upregulation in tumors. 

Because of the established roles that both CA IX and CA XII play in the process of tumorigenesis, cancer cell signaling, tumor progression, acidification, and metastasis, many classes of CA IX/CA XII-targeted inhibitors and biologics have been studied in the preclinical setting. These studies have yielded promising results showing that inhibition of CA IX/CA XII catalytic activity decreased the growth, proliferation, and metastatic potential of several aggressive cancers both in vitro and in vivo [36,52,53,54,55]. The most successful to date include the use of sulfonamide-based compounds and monoclonal antibodies for the treatment of cancers that overexpress CA IX or CA XII. Please see the extensive reviews published by us and others [32,50,56,57]. Two of the sulfonamide-based inhibitors (SLC-0111 and E7070/Indisulam) are currently in clinical trials. Clinical trials involving immunotherapy using monoclonal antibodies (G250 and its chimeric derivative cG250) alone or combined with other therapeutic techniques are also currently under development [58,59,60,61]. In addition, immuno-detection strategies have also been adopted to target CA IX for molecular imaging of tumor hypoxia [62,63,64]. The goal of detecting hypoxia in a non-invasive manner, could predict patient outcome to drug therapy and serve as a tool to inform treatment decisions.

In this review, the current understanding of CAs role in tumor physiology and pH regulation is discussed. The expression and function of each CA isoform in both normal and tumor cells/tissues, as well as some of the commonly found mutations in tumor specimens, will be discussed. The goal of this review is to provide a comprehensive overview of the CA family and their combined role in cancer and current anti-cancer therapies.

## 2. pH—The Role of CAs in Tumors

### 2.1. Differential pH Creates the Ideal Conditions for Tumor Cell Proliferation and Survival

Primary tumors are often described as heterogeneous, in that cells of different types, metabolic states, and stages within the cell cycle can exist at any given time-point [65,66]. This diversity complicates anti-cancer treatment targeting the primary tumors, and becomes even more complicated as tumors reach later stages and/or become highly aggressive. One of the most problematic situations occurs in the context of hypoxia. Tumor hypoxia has been well characterized and is initiated through environmental or genetic factors causing a metabolic shift towards rapid aerobic glycolysis [67,68]. This process is commonly known as the “Warburg Effect” and is defined by increased glucose consumption via glycolysis diverting glucose carbons to lactic acid, even in the presence of oxygen [69,70]. This limits ATP production via oxidative phosphorylation but increases ATP production by glycolysis [71]. There is evidence that oxidative phosphorylation is supported by anepleurotic reactions particularly through glutamate dehydrogenase, which converts glutamate (derived from glutamine) to α-ketoglutarate another commonly observed metabolic alteration in cancer [72,73,74]. Additionally, glycolysis supports the synthesis of phospholipids by providing the glycerol phosphate backbone via the glycolytic intermediate, dihydroxycetone phosphate. This is important for membrane biogenesis, which underlies the success of cancer cell replication [75]. When tumor cells transition to a hypoxic, or the aerobic glycolytic state, there is a measurable pH difference between the extracellular and intracellular pH (pH_e_ and pH_i_, respectively). In part, this is postulated to be due to the over production of lactate because of high glycolytic rates and inhibition of pyruvate decarboxylation in the mitochondria. Export of metabolic acids ultimately lowers pH_e_ [76]. 

In most normal cells, a pH differential is maintained between pH_i_ and pH_e_ such that the extracellular space maintains a slightly more basic environment (pH_e_ ≥ 7.3) relative to the intracellular environment (pH_i_ = 7.2) [77,78,79]. This gradient permits the function of normal metabolic, transport, and regulatory processes. In hypoxic tumor cells, however, pH_e_ drops to values ranging from 6.5–7.1 with only a marginal decrease in pH_i_ (≥ 7.2) [77,78,80]. This activates a cascade of events that provide an advantage for tumor cell survival and proliferation. Specifically, the acidic pH_e_ becomes favorable for extracellular matrix (ECM) remodeling, limits HCO_3_^−^ dependent dynamic buffering, and induces acid activation and expression of proteases, resulting in the facilitation of tumor cell dissemination and invasion. Additionally, the slight decrease in pH_e_ favors tumor cell proliferation, metabolic adaptation, migration pathways, and results in evasion of apoptosis. This ultimately sets up conditions that benefit tumor cell survival and proliferation, resulting in an unfavorable prognosis for cancer patients [67,76,78,81]. The increase in pH_i_, when compared to the more acidic pH_e_, favors flux through glycolysis and inhibition of gluconeogenesis (mostly in the liver and pancreas) [82,83]. Specifically, pH_i_ ≥ 7.2 stimulates lactate dehydrogenase (LDH) activity, which has an in vitro pH optimum of ~pH 7.5. This enzyme mediates the conversion of pyruvate to lactate and regenerates NAD^+^, which is required for continued glycolytic activity [84,85]. Furthermore, the increased pH_i_ increases expression of several glycolytic enzymes, thus contributing to the high rate of observed glycolytic activity within the tumor cell. Alternatively, a lower pH_i_ (<7.2) will reverse these conditions and decrease expression of glycolytic enzymes such as LDH, and transporters like GLUT1 [86,87]. 

The decrease in pH_e_ is caused by the rapid extrusion of lactic acid and free protons from tumor cells, resulting from the upregulation of glycolysis in the cytosolic compartment. It has been postulated that glycolytic enzymes cluster at the inner surface of cell membranes and interact with ion and proton transporters at the cell surface. This allows for a rapid transport of protons both in and out of the cell depending on the shift in metabolic equilibrium achieved within the cellular microenvironment [88,89]. In addition, an acidic pH_e_ establishes a favorable environment for cell metastasis and invasion [90]. Specifically, acidic pH_e_ enhances expression and activities of ECM reorganizational proteases, such as matrix metalloproteinases (MMPs) and cathepsin B [91,92]. In combination with this, an increased pH_i_ ≥ 7.2 creates an environment that favors de novo actin filament formation through the expression and activation of actin-binding proteins such as cofillin, villin, profilin, twinfilin, and talin [93,94,95,96,97,98]. This, in turn, promotes metastatic and invasive tumor cell behavior [93,94,95,96,97,98].

This unique pH profile in tumor cells also permits cancer cell proliferation through bypassing cell cycle check points and evasion of apoptotic pathways [99,100]. When pH_i_ ≥ 7.2, there is an increase in the activity of CDKs, specifically CDK1, which increases the efficiency of MAPK pathways [101]. This stimulates the rate of progression through the G2/M phase and into the S phase, where tumor cells become more adapted to proliferation and less sensitive to chemo- and radiation therapies [101]. In addition, the increased pH_i_ suppresses DNA damage checkpoints that would typically slow the progression of a cell through the G2/M phase and restrict proliferation [78]. In combination with this, a pH_i_ ≥ 7.2 favors a suppression of apoptotic pathways [100,102]. In normal cells (where pH_i_ = 7.2), a reduction in pH_i_ to < 7.2 would result in a conformational change in the pro-apoptotic factor, BAX, causing its activation and interaction with the mitochondrial membrane [103]. This interaction causes the release of cytochrome c from the inner mitochondrial membrane and activation of other pro-apoptotic factors such as the caspases [100]. Caspase activity achieves optimal efficiency near pH 6.8 in vitro. When the pH_i_ becomes slightly more alkaline, these pathways are suppressed, allowing the tumor cells to resist apoptosis [100]. In addition, with a pH_i_ ≥ 7.2, which is the case in most tumor cells, there is a high probability that this anti-apoptotic pH level will be maintained, even in cases where there may exist a small influx of protons. Taken together with pH-induced ECM and metabolic transitions, it is clear that the unique pH differential across membranes drives tumor cell proliferation and survival. 

### 2.2. CAs’ Role in Creating a Tumor Cell pH Differential 

If we can understand the complex factors that establish the unique pH environment of tumor cells, it may be possible to develop therapeutics strategies that can reduce or inhibit these factors to re-normalize pH. In recent years, the carbonic anhydrase family has been shown to create and maintain the pH differential in tumor cells [104]. Evidence over the last two decades has determined CAs play a pivotal role in tumor cell metabolism, migration and invasion, and also in cell survival [3,50,105]. This has prompted efforts by several groups to determine targeting strategies against CAs to inhibit cancer progression. Despite the progress that has been made, there remains many unanswered questions about the role CAs play in tumor cell biology, and the mechanistic details that correlate CA inhibition with the observable therapeutic effects on cancer. 

It is widely accepted that CAs are the driving force in pH regulation in primary tumor cells as they are for normal tissue. These enzymes function, through their hydratase/CA activity, to regulate the production of bicarbonate, the universal physiological buffer. Along with this, CAs produce or sequester protons. In addition to their hydratase activity, CAs also have a slower esterase activity, which is mediated by the same catalytic pocket with a mechanism similar to that of the hydratase/CA activity [106]. Thus, many investigators use esterase activity as an indicator of the hydratase activity [106,107]. The role of this esterase reaction in cancer, however, is currently unknown. The two-step CA reaction mechanism is given below (Equations (1) and (2)), and reviewed in detail by Lindskog and Coleman [108], and again by Frost and McKenna [3]. The process, common to reversible biochemical pathways, can be thought of as a two-step equilibrium, whereby substrate/product concentrations determine the reaction direction. In most normal tissues, it is predicted that this results in a constant supply of buffering agents for the maintenance of physiological pH. How does this process contribute to distinct regulation in the hypoxic tumor cell? Two hypotheses have been proposed to describe how CA activity can regulate and foster the unique pH differential within a tumor cell.

(1) $${\mathrm{EZn}}^{2+}-{\mathrm{HO}}^{-}+{\mathrm{BH}}^{+}\Leftrightarrow {\mathrm{EZn}}^{2+}-H_{2}O$$

(2) $${\mathrm{EZn}}^{2+}-H_{2}O+{\mathrm{HCO}}_{3}^{+}\overset{H_{2}O}{↔}{\mathrm{EZn}}^{2+}-{\mathrm{HCO}}_{3}^{-}\Leftrightarrow {\mathrm{EZn}}^{2+}-{\mathrm{OH}}^{-}+{\mathrm{CO}}_{2}$$

**Figure 1:**
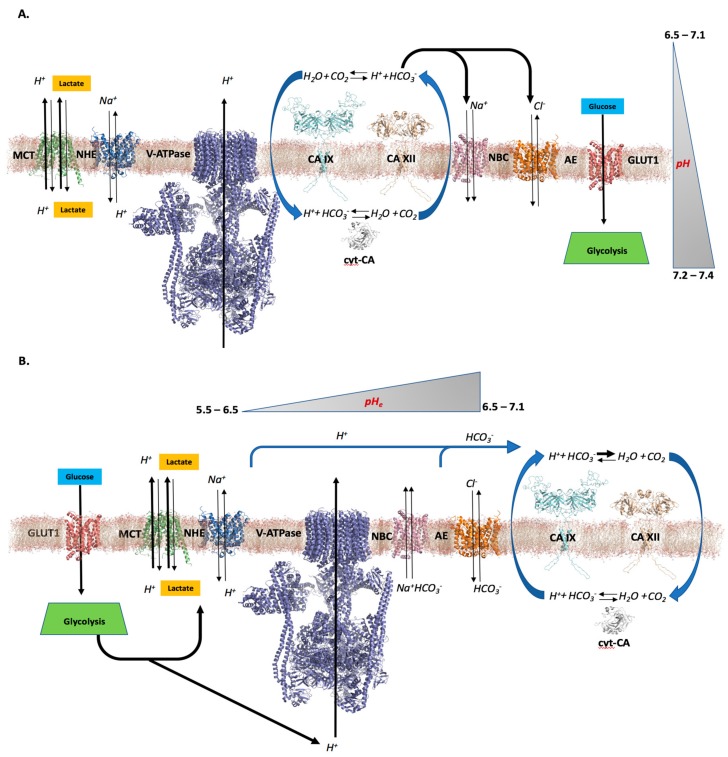
Illustrations of Hypothesis 1 (**A**) and Hypothesis 2 (**B**) to model the functional role of CA in the hypoxic tumor microenvironment. (**A**) Hypothesis 1: CA IX and XII (cyan and tan, respectively) are located on the extracellular membrane and adjacent to transporters involved in pH regulation. Here, CA IX and XII act in conjugation with cyt-CAs (grey) to cycle substrates of water, CO_2_, HCO_3_^−^, and protons, to maintain the differential pH microenvironment. (**B**) Hypothesis 2: Extracellular CA IX and XII act to raise pH_e_ that has been reduced to levels < 6.0 due to excess expulsion of glycolysis byproducts. In this case, HCO_3_^−^ dehydration and proton sequestration are catalytically favored by CA IX and XII. Alternatively, cyt-CAs act to convert excess CO_2_ to HCO_3_^−^ to buffer pH_i_ and provide substrates to be transported to the extracellular surface to be utilized by CA IX and XII. Transporters shown are MCT (green), NHE (blue), V-ATPase (purple), NBC (pink), AE (orange), and GLUT1 (red). Structural models were generated using PyMol [119]. Models of CA IX and XII were generated using PDBs 5DVX [51] and 1JCZ [120], respectively. PDB 3J9T was utilized to model V-ATPase [121], and PDB 4YZF [122] was utilized to generate models of AE, NBC, and NHE. For modeling of MCT and GLUT1, PDBs 1PW4 [123] and 4PYP [124] were used, respectively. To represent cyt-CAs, PDB 3KS3 [125] was utilized.

**Hypothesis** **1** **(Figure** **1A).** *This hypothesis suggests cooperative activity between cytosolic (such as CAs I and II) and extracellular CAs (IX, XII, and perhaps XIV) that ‘cycle’ substrates for pH regulation between the extracellular and intracellular tumor environment. Specifically, extracellular CAs take advantage of the large quantity of CO_2_ present in the extracellular space, and convert this to HCO_3_^−^ and protons. Adjacent ion transporters, such as anion exchangers (AE1–AE3), can then traffic HCO_3_^−^ [109] into the cytosol while the free protons remain outside the cell thus lowering the pH_e_. Inside the cell, the newly imported HCO_3_^−^ can be converted back to CO_2_, which can be used in metabolic pathways or diffuse back outside of the cell carrying that sequester proton. This restores pH_i_ to more normal levels (Figure 1A) [30,57,110]. This pathway relies on several assumptions. First, extracellular CA activity must remain active at both an alkaline pH of 7.4 and as the pH_e_ decreases (<6.5). Originally, it was proposed that CA IX, one of the most prevalent extracellular CAs in hypoxic tumors, retained its activity in acidic and basic environments through the use of an ‘internal buffer’, which exists as an extended N-terminal domain, called the PG domain [111]*. The PG domain of CA IX has a large quantity of aspartate residues* [111]. It is thought that these residues could act as titratable proton sinks that prevent protons from interacting with the active site of the enzyme and therefore allow it to retain activity in acidic environments [111,112]. Recent evidence has determined that the PG domain is not necessary for the enzyme to retain activity in acidic conditions, as the catalytic domain alone of CA IX can retain activity in pH as low as 5.0 [51].*

The above hypothesis also relies on the ability of CA IX (or other membrane-associated CA) to cluster close enough to bicarbonate transporters, such that newly synthesized HCO_3_^−^ can be readily transported into the cell rather than diffuse away. This is supported by recent evidence showing that glycolytic enzymes, in association with CA, cluster near transporters at the cellular membrane [89,113,114,115]. However, for this hypothesis to be valid in hypoxic tumors, it would also have to account for the protons produced by extracellular CAs, and a rationale for how these free protons are not rapidly transported into the cytosol given there is an established intra/extracellular proton concentration gradient and the reversible nature of some transporters. It is possible that there is a significant difference in proton concentration between the intra- and extracellular space, where both transporters and glycolytic enzymes are clustered, and as a result, proton expulsion is favored rather than uptake into the cell. This would account for the observed pH differential within the tumor microenvironment and further reinforce the feasibility of this hypothesis depicting the role CA plays in hypoxic tumor cells. 

Another factor that must be taken into account is extracellular lactate concentration and its effect on CA IX activity. In 2001, Innocenti et al. showed that CA IX has low sensitivity to inhibition by lactate (K_i_ > 150 mM) representing an evolutionary adaption of the enzyme to harsh conditions such as high lactate levels, increased acidity and hypoxia [116]. It is also interesting to note that pyruvate, the oxidized analog of L-lactate, is a CA IX inhibitor (K_i_ of 1.12 mM) more potent than HCO_3_^−^ (K_i_ of 13 mM) where as its reduced form, under hypoxic conditions, is not [116]. These data suggest an evolutionary adaptation of CA IX to assure an efficient CO_2_ hydration activity in tumors exposed to more harsh conditions than in the physiological setting.

**Hypothesis** **2** **(Figure** **1B).** This hypothesis is similar to Hypothesis 1, in that both extracellular transmembrane CAs and cytosolic CAs play a role in tumor cell pH regulation. However, in this alternative hypothesis, it is proposed that extracellular CAs do not necessarily lower pH_e_ and produce substrate (HCO_3_^−^) for cytosolic CAs, but instead buffer pH_e_ to levels (between 6.5–7.1) that promote tumor cell survival and avoid tumor necrosis. In this case, the tumor cell has begun to transition to a hypoxic state and its metabolic shift toward glycolysis has taken place. This causes a rapid extrusion of lactic acid and a drastic reduction in pH_e_, which has been recorded at levels as low as 5.5. At this pH, the cell would soon undergo necrosis resulting in its death. But we know that cancer cells continue to survive and proliferate within the acidic microenvironment. It is postulated that the extracellular CAs act to sequester protons in the form of CO_2_ to raise and maintain the pH_e_ to a level that favors tumor cell growth, proliferation, and survival (Figure 1B). This has been postulated by work from Li et al. [33] *and Mahon et al.* [51] These studies showed that extracellular CA activity, especially CA IX, from both purified enzyme and in membranes isolated from MDA-MB-231 cells (which display hypoxia-dependent CA IX upregulation), favors HCO_3_^−^ dehydration at lower pH. Thus, CA IX under proton load will sequester protons through its catalytic activity in the form of CO_2_ in the extracellular space, which increases pH_e_. Together, these studies have shown that CA IX is able to both retain activity at low pH levels (pH < 5.0) and preserve the core, folded structure (as low as pH = 2.0). Both of these attributes are necessary for the enzyme to stabilize pH_e_ in an acidic environment. Sweitach et al. has also suggested that the net effect of CA IX on pH_e_ will depend on the emission of CO_2_ relative to lactic acid [117]. In their studies, CA IX was shown to be important in the formation of extracellular and intracellular pH gradients in multicellular spheroid growths of cancer cells [117]. When CA IX expression was reduced, spheroids developed very low pH_i_ (~6.3) and pH_e_ was measured at pH ~ 6.9 at the core [117]. However, with CA IX expression, an increase in pH_i_ (~6.6) was observed and extracellular acidity was increased (pH_e_ ~ 6.6) [117]. While these data appear to support Hypothesis 1, the authors conclude that the activity of CA IX can reduce pH_e_ but the direction of the reaction is ultimately dependent on substrate availability, i.e., lactate levels and the pH of the tumor microenvironment.

### 2.3. Further Considerations

**Figure 2:**
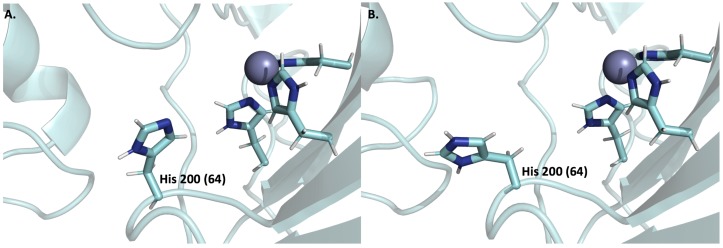
Positions and protonation states of CA IX catalytic His 200 (64 with CA II numbering) that has been implicated in proton sequestration in the acidic tumor microenvironment. (**A**) Deprotonated His 200 (PDB: 4Q49) in an “in” position at neutral pH = 7.5. (**B**) Protonated His 200 (PDB: 4Y0J) in an “out” position at acidic pH (pH = 6.0) representative of a “proton sequestered” state as in the acidic tumor microenvironment. Neutron crystallography structures of CA II were used to determine protonation states and positions and were previously determined by Fisher and McKenna [126].

Recent evidence found by Jamali et al. [68] has suggested that extracellular CA acts to sequester protons via its catalytic histidine residue (200 using CA IX numbering, commonly referred to as His 64 using CA II numbering). Specifically, the study finds that a single monomer of an extracellular CA can sequester a proton via residue (Figure 2). This result favors Hypothesis 2, whereby extracellular CAs act more favorably to raise pH_e_ in tumors. Furthermore, this result suggests that the presence of the PG domain of CA IX has limited involvement in pH regulatory processes, in contrast to previous thought. If it is true that extracellular CA IX acts to regulate pH through sequestering a single proton via a His residue then several questions arise: (1) what is the maximum pH_e_ regulating potential of all extracellular CAs on the tumor cell surface? And (2) is this significant enough to contribute to the pH differential observed in tumor cells, which would validate this hypothesis? To answer these questions, the pH regulating potential, or change in pH_e_ (∆pH_e_), induced by an extracellular CA acting as a proton-sequestering agent can be estimated. Consider that the concentrations of CA IX and/or XII dimer (common extracellular CAs in tumor cells) is ~19 nM for a single hypoxic tumor cell with a diameter = 20 µm and an initial pH_e_ of 6.5. In addition, assume that a monomer of extracellular CA can sequester protons in a ~1:1 ratio via a single active site histidine. With these assumptions, the following expression can be used to establish the relationship between pH and proton concentration in a solution:

(3) $$\Delta pH_{e}\;=\;-log\left({\left[H^{+}\right]}_{tot}-n\left[CA\right]\right)-pH_{e}.$$

Here, ${\left[H^{+}\right]}_{e}$ represents the concentration of total protons in the extracellular environment, n[CA] represents the CA concentration multiplied by an integer that is equivalent to the CA oligomeric state (considering CA IX and XII are dimers, this would be n= 2), and pH_e_ represents the initial pH of the extracellular environment (in this case is 6.5). Considering only these parameters, the total quantity of extracellular CA in a single tumor cell contributes an overall $\Delta pH_{e}$ of 0.05 units over time. Considering that a change in pH of ~0.1 units in cellular environments can drastically change a cell’s behavior and functions [118], this contribution becomes significant. Of course, this also indicates, as predicted, that other factors also contribute to the pH differential and regulatory processes in tumor cells. It should also be emphasized that this is an approximate estimation and relies on several assumptions that may not fully reflect the tumor cell at any given time. However, it allows us to consider the maximum effect that these CA-proton sequestration processes may have on tumor cell pH regulation. 

It is also important to note that CAs catalyzes a reversible reaction and this reaction depends on substrate/product availability. Hypothesis 2 takes this bi-directionality into account, hence the buffering capacity of extracellular CAs. For instance, at pH values lower than ~6.8, CA IX has been shown to favor its dehydration reaction (H^+^ + H_2_CO_3_^−^ → CO_2_ + H_2_O) over hydration (CO_2_ + H_2_O → H^+^ + H_2_CO_3_^−^), while the opposite is expected to happen at much higher pH [33]. This evidence supports the notion that membrane bound CAs, especially CA IX (pKa of Zn-H_2_O ~ 6.3), work not only to prevent the unfavorable acidification of the tumor microenvironment, as a result of the metabolic switch, but to “adjust” the pH_e_ to favor tumor growth, progression, and eventually metastasis. The role of cytosolic CAs in both hypotheses remains similar. Cytosolic CAs will utilize available substrates in the form of CO_2_, water, HCO_3_^−^, and protons, allowing them to buffer the intracellular environment to maintain the slightly alkaline pH that is observed. 

It is possible that each of these hypotheses in defining the role of CAs in the differential pH environment of a tumor cell have validity. For instance, the fate of extracellular CO_2_ converted to HCO_3_^−^ can be rationally explained by Hypothesis 1, particularly at the initiation of tumor growth where hypoxia is not an issue, as this explains the location of CO_2_ and HCO_3_^−^ both in the intra- and extracellular environment. Alternatively, the process of proton sequestration and the role of extracellular CA activity in reducing and maintaining pH_e_ is more rationally explained by the Hypothesis 2, particularly with the support from recent experimental evidence [33,51,113]. Of course, more experiments will be needed to address the strength and weaknesses of these hypotheses in explaining the role of CA in tumor cells, which will drive a more accurate model of the function of these enzymes in the microenvironment. This becomes more important as the effort toward creating CA targeting anti-cancer therapies increases and as more compounds progress through clinical trials. 

## 3. Cytosolic CA Isoforms: Expression, Distribution, and Function

The eight active cytosolic CA (cyt-CA) isoforms include CA I, II, III, VII, VIII, X, XI, and XIII [3,48]. Because there is limited knowledge of the roles CA-RPs play in terms of normal or tumor physiology, they will not be further discussed in this review. The cyt-CA isoforms have >50% primary sequence identity (Table 1). The greatest number of conserved residues are between CAs I and II, both of which are highly expressed in red blood cells (Table 1) [127,128,129]. The cyt-CAs are ubiquitously distributed in human tissue and show diverse functional roles despite having conserved enzymatic activity. Expression of cyt-CAs has been observed in red blood cells, kidneys, skeletal muscle, adipose tissue, colon liver, brain, and neurons [39,127,128,129,130,131,132]. Primarily, functions of cyt-CAs include maintaining physiological pH of the blood through production of HCO_3_^−^, often through interactions with transporters to facilitate efficient HCO_3_^−^/proton flux together they are called transport metabolons [133,134,135]. In addition, cyt-CAs contribute to maintaining normal cell metabolism and also show involvement in neuronal excitement and signaling pathways [135]. Interestingly, specific cyt-CA function correlates with tissue distribution.

### 3.1. Expression and Function of CA I and CA II in Normal Cells

High levels of cyt-CAs I and II expression are found in red blood cells and are necessary for maintaining physiological pH of the blood. It is suggested that CA II activity is the more dominant because of its greater expression and increased catalytic efficiency (in vitro) compared to CA I, although the intracellular environment might dictate how these enzymes behave in vivo (Table 2). In addition to red blood cells, both CA I and II are expressed in the GI tract, lungs, bone marrow, the eye, and also in the extracellular exosome-enriched fraction from normal human urine [136,137,138,139]. On its own, CA II expression has been observed in kidney, liver, brain, salivary gland, testis, and minimal expression observed in breast tissue [137,138,139,140]. CA II function is essential for bone resorption and osteoclast differentiation, and for regulation of fluid secretion into the anterior chamber of the eye. Recent evidence has implicated CA II activity in association with and activation of several ion transporters, which suggests that CA II acts as mediator of certain metabolic pathways by providing additional substrates for the transporters to balance cytosolic pH. As a result of these interactions, CA II is associated with several diseases including glaucoma, renal tubular acidosis, cerebral calcification, cardiomyocyte hypertrophy, growth retardation and osteoporosis [31,130,131,132]. Abnormal levels of CA I expression in the blood are a marker for hemolytic anemia [131]. Taken together, these show that in comparison to CA I, CA II expression is abundant in normal tissues and consequently has well-established and important physiological functions. 

### 3.2. Expression and Function of CA I and CA II in Tumor Cells

The potential of CA I as a tumor-associated isoform has not been extensively studied. However, according to multiple genomic databases including the cBioPortal of the cancer genome atlas (TCGA) and the human protein atlas, medium to high levels of CA1 mRNA was detected in acute myeloid leukemia, colorectal cancer, and renal carcinoma patients, based on RNA sequencing [136,139,141,142]. Immunohistochemical (IHC) staining of malignant tissues showed strong cytoplasmic and nuclear CA I staining in a few lymphomas and medium to high staining in renal cancer, melanomas, and stomach cancers [136,138]. Recent studies have also shown that CA I contributes to mammary microcalcification, tumorigenesis, and migration in breast cancer [143]. Furthermore, high CA I expression was observed in the sera of stage I non-small cell lung cancers (NSCLC), suggesting that CA I can be used as a potential biomarker for early detection of NSCLC [144].

**Figure 3:**
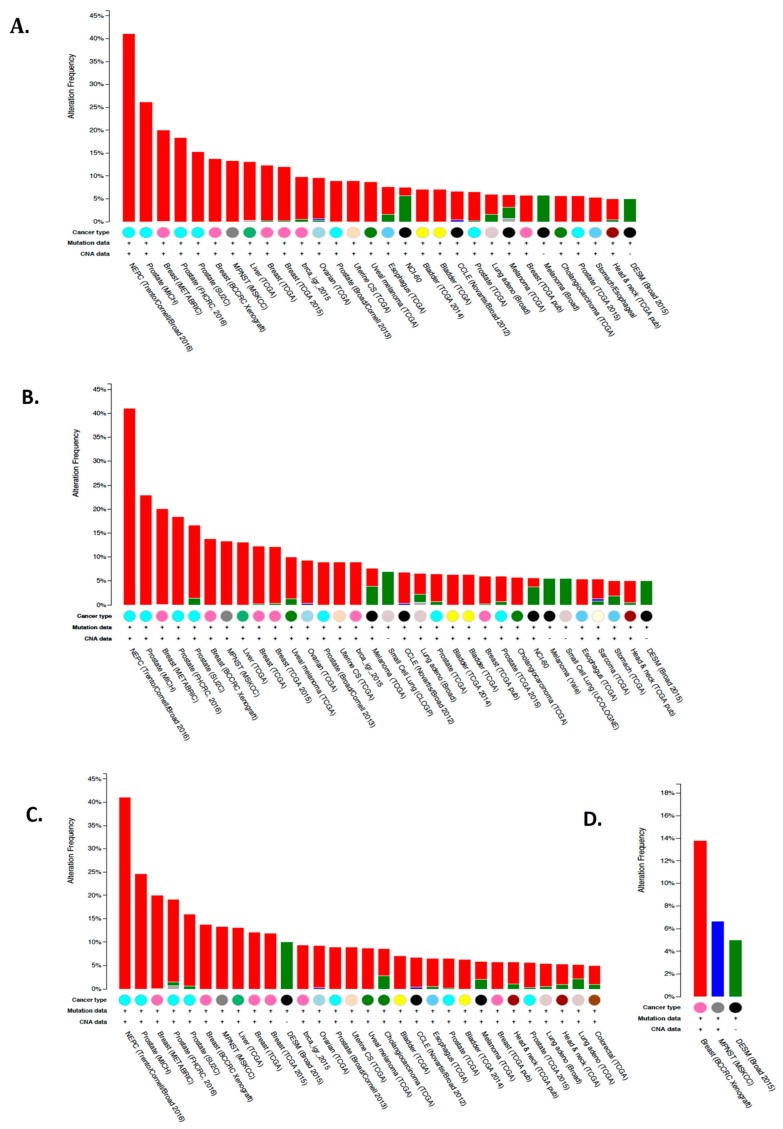
Summary of cross-cancer alterations for CA isoforms. (**A**) CA I, (**B**) CA II, (**C**) CA III, and (**D**) CA VII. Gene amplifications are shown in red, mutations in green, deletions in blue, and multiple alterations in gray. The minimum percent altered samples threshold was set to 5%. The data and results were obtained from the cBioPortal of the human genome atlas (TCGA) [141,142]. Experimental details of each study referenced can also be found in the TCGA database.

CA I has also been shown to be upregulated in human pancreatic cancer (PDAC) where its expression correlated with tumor de-differentiation. Biopsies of patients with PSA positive gray-zone levels (serum prostate-specific antigen levels ranging from 4 to 10 ng/mL is considered a diagnostic gray zone for detecting prostate cancer) also tested positive for plasma CA I [145]. This suggests that CA I can potentially serve as a plasma biomarker, and the combination of PSA and CA I detection may have great advantages for diagnosing prostate cancer in patients with gray-zone PSA levels [145]. The most common alterations in cancer for the CA1 gene are amplifications, as high as 40%, in neuroendocrine prostate cancer, prostate adenocarcinomas and metastatic cancers [141,142]. Recently, CA1 gene amplification was detected in approximately 25% of breast cancer studies [141,142]. Other gene alternations include mutations (missense and truncations) and gene deletions. The most frequent mutations were observed in the mRNA of breast, lung and melanoma patients (Figure 3A) [141,142]. However, it is currently unknown if these mutations result in changes in gene expression or affect activity.

Unlike CA1, detection of CA2 mRNA expression using RNA sequencing showed a more widespread upregulation in cancers. These include but are not limited to prostate, melanomas, bladder, thyroid, breast, lung, liver, pancreas, gliomas (with the highest expression observed in glioblastomas), renal cell carcinomas, and head and neck cancers [141,142]. Like CA1, the most common gene alterations in CA2 were amplifications especially in neuroendocrine prostate cancer, breast cancer, prostate adenocarcinomas and metastatic cancers followed by mutations (Figure 3B) [141,142]. This infers an increase in expression and activity, although this has not been measured. IHC showed strong cytosolic staining in gastric, pancreatic, and cervical cancers, and medium staining in breast, renal, and liver cancers [136,137,138,140]. As a biomarker, low CA II protein expression is often associated with tumor aggressiveness and poor prognosis in some cancers including pancreatic ductal adenocarcinomas (PDAC), colorectal, gastric and gastrointestinal stromal cancers [35,146,147,148]. Therefore, this isoform can be considered both a diagnostic and an independent prognostic factor for favorable outcome and overall survival in the aforementioned cancers. However, in other cancers (such as astrocytomas, oligodendrogliomas, melanomas, pulmonary endocrine tumors, and breast cancer) CA II upregulation is associated with poor prognosis, tumor progression, and metastasis [146,149,150,151]. Thus both CAs I and II may be potential targets for the treatment of many cell-type specific cancers, Because we are in the early stages of developing targeting strategies, care should be taken not to overestimate the role of CA I and CA II in the regulation of pH and tumor growth, nor as prognosticators or targets, until these strategies, are further documented. 

### 3.3. Expression and Function of CA III and CA VII in Normal Cells

In normal cells, CA III expression has been detected in both skeletal muscles and adipose tissues (both white and brown fat), with medium expression also observed in the breast [136,137,152]. CA III displays a 200-fold decrease in catalytic activity compared to CA II and is considered the slowest isoform in terms of its catalytic activity (Table 2) [48]. This difference in activity has led to the hypothesis that CA III might serve different physiological roles unrelated to its primary catalytic function. These include gene regulation, adipogenesis, metabolism, and protection in response to oxidative stress. Recent studies have also shown high CA III expression in osteocytes where its expression is regulated by parathyroid hormone both in vitro and in vivo. In that capacity, it functions to protect osteoclasts from hypoxia and oxidative stress [153]. CA VII, which is one of the least characterized CA family members, is expressed primarily in the colon, liver, skeletal muscle and brain. It has the highest esterase activity among the CA family members. CA VII, like CA III, has been suggested to play a role as an oxygen free radical scavenger, because of the presence of two reactive cysteines that can be glucothionylated. CA VII has also been implicated in neural excitation, seizures and in that regard may represent a drug target for treatment of seizures and neuropathic pain [3,4].

### 3.4. Expression and Function of CA III and CA VII in Tumor Cells

There are few published studies that have focused on the expression and function of CA III and CA VII in tumors. However, according to the RNA sequencing and IHC data deposited to the TCGA and human protein atlas respectively, CA3 mRNA transcripts were observed in multiple cancers, with the highest median expression in glioblastomas and thyroid cancers [136,137,138,139,141,142]. Although no CA III mutations in the aforementioned cancers were described, mutations were observed in lung, melanomas, and head and neck cancers and the most common mutations in these cancers include missense mutations (Figure 3C). Since two of the aforementioned cancer types are high among smokers, these mutations may be attributed to smoking. Ultraviolet damage from the sun may also occur in melanoma patients, although still speculative at this point. Furthermore, IHC showed strong cytoplasmic and nuclear staining in one study of renal cancer along with a few basal cell carcinomas of the skin [136,139]. Like CAs I and II, the most frequent alterations observed were gene amplifications, which occurred in prostate and breast cancers [141,142]. 

The median expression for CA7 mRNA transcript in tumors is much lower than the previously mentioned isoforms, with the highest expression observed in thyroid carcinoma, colorectal adenocarcinoma, and lower-grade brain gliomas [141,142]. In the latter, CA VII upregulation may act as a marker for poor prognosis. The most frequent gene alterations are also different from the previously mention cytosolic isoforms, because they are low in abundance and are more varied from tumor to tumor (Figure 3D). The highest alteration, which is CA7 amplification, was observed in breast cancer patient xenografts followed by deletions in malignant peripheral nerve sheath tumors and then mutations in desmoplastic melanoma (Figure 3D) [141,142]. IHC staining of CA VII showed weak to moderate cytoplasmic and occasional nuclear expression [136]. However, a few cases of ovarian and gastric cancers exhibited strong staining [136,139]. 

A study published by Kuo et al. discovered reduced levels of CA I, II and III in human hepatocellular carcinoma (HCC) compared to adjacent normal tissue. In 2008, a study showed that CA III expression promotes the transformation and invasive capacity of hepatoma cells through the focal adhesion kinase (FAK) signaling pathway [154]. In this study, it was hypothesized that CA III is re-expressed in later stages of metastatic progression of HCC, and it might have an important influence in the development of metastasis in liver cancer [154]. Since then, no other studies have been published specifically looking at CA III expression and function in cancer. A recent study has, however, shown that CA VII expression has some prognostic value in colorectal carcinoma (CRC) [155]. CA VII expression was frequently downregulated in CRC tissues at both the mRNA and protein levels. Decreased expression of CA VII was significantly correlated with poor differentiation, positive lymph node metastasis, advance TNM (T-refers to the tumor size, N-refers to ‘node’ status and M-refers to ‘metastasis’) stage and unfavorable clinical outcome [155]. This suggests that CA VII can be used an independent prognostic indicator for patients with early stage CRC, and CA III may serve as a therapeutic target in the treatment of metastatic liver.

### 3.5. Expression and Function of CA XIII in Normal and Tumor Cells

Human CA XIII isoform was first identified and characterized in 2004 [28,156]. It is considered a slow isoform in terms of its catalytic activity (1.5 × 10^5^) comparable to CA I (Table 2). CA XIII expression is observed in several tissues including kidney, brain, lymph nodes, thyroid, liver, GI tract, skin, adipose, soft tissue, and in both male and female reproductive organs [39,136,138]. It has been hypothesized that CA XIII plays a role in pH regulation of reproductive processes including maintenance of sperm mobility and the normal fertilization process. To date, no direct proof for a significant physiological function for CA XIII has been reported. However, downregulation of CA XIII has been seen in cases of colorectal cancer and the lowest signal was detected in carcinoma samples, although the clinical significance of these observations is yet to be determined [39,136,139].

**Figure 4:**
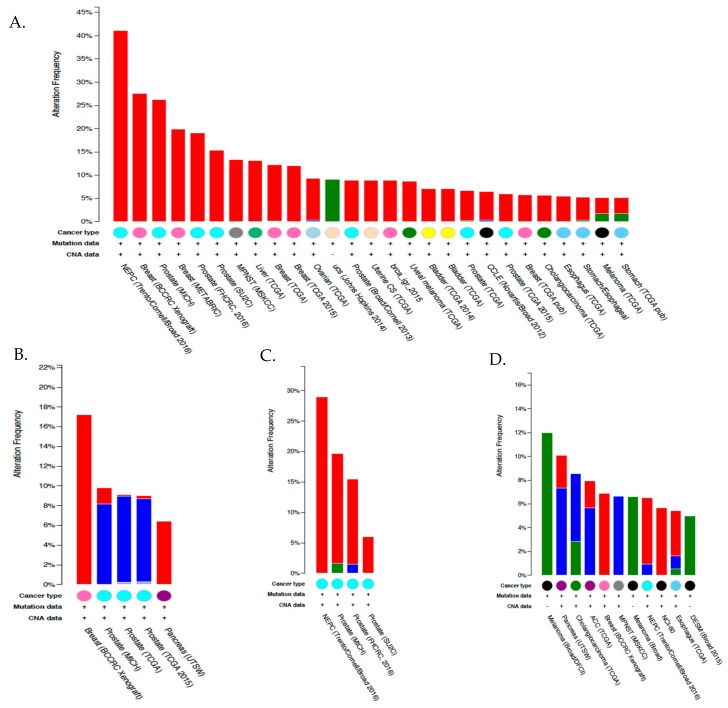
Summary of cross-cancer alterations for CA isoforms. (**A**) Cytosolic isoform CA XIII, (**B**,**C**) mitochondrial isoforms CA VA, and CA VB, respectively, and (**D**) secreted isoform CA VI. Gene amplifications are shown in red, mutations in green, deletions in blue, and multiple alterations in gray. The minimum percent altered samples threshold was set to 5%. The data and results were obtained from the cBioPortal of the TCGA database [141,142]. Experimental details of each study referenced can also be found in the TCGA database.

Nonetheless, the downregulation of cytosolic CA I, II and XIII in colorectal cancer may result from reduced levels of a common transcription factor or loss of the closely linked CA1, CA2 and CA13 alleles on chromosome 8. According to IHC data submitted in the human protein atlas, most cancer tissues showed weak to moderate CA XIII immunoreactivity. However, strong staining was observed in renal and pancreatic cancers. Most thyroid cancers, several gliomas, gastric, and liver cancers showed weak to moderate cytoplasmic staining, while melanomas, lung, and skin cancers were only weakly positive [136,138]. Similar to the IHC data, CA13 mRNA expression was most highly upregulated in thyroid, RCC, lung, pancreas but additionally colorectal and testicular germ line cancers [141,142]. The most common alteration, however, was gene amplification, which was observed in prostate and breast cancers (Figure 4A). Although not much attention has been given to CA XIII with regard to cancer because of its high expression in normal cells, it could have some prognostic and diagnostic value. 

## 4. Mitochondrial CA Isoforms: Expression, Distribution, and Function

The first mitochondrial α-CA isoform discovered and isolated from guinea pig liver was CA V [25]. Two different transcripts of this isoform were later identified and coined CA VA and CA VB [26,27,157]. These two transcripts have 59% primary sequence identity and 184 conserved residues (Table 1). This number is slightly lower than that observed between CA isoforms I and II. Although, the tissue-specific distribution pattern between the two is significantly different, CA VA and CA VB are the only two isoforms exclusively expressed in the mitochondrial matrix of hepatocytes and adipocytes, respectively [157]. Interestingly the human ortholog for CA5B, which has broad tissue expression, has been mapped to chromosome Xp22.1, while CA5A was mapped to 16q24 [25,26]. Their roles within specific tissues include ureagenesis and lipogenesis but may also serve as mediators in several other metabolic pathways as discussed below. These functions suggest that both enzymes could be considered as anti-obesity and anti-diabetic drug targets, studies of which are currently being pursued [158,159,160,161]. 

### 4.1. Expression and Function of CA VA and CA VB in Normal Cells

Mitochondrial isoform CA VA has been shown to be directly associated with ureagenesis [3,4,162]. CA VA produces bicarbonate, which is a substrate for carbamoyl phosphate synthetase I in the synthesis of carbamoyl phosphate, the rate-limiting step of ureagenesis [162]. Bicarbonate production by CA VA can also drive other biosynthetic reactions like that of pyruvate carboxylase, which mediates an important anepleurotic step in the Krebs cycle from which substrates can be drawn for biosynthetic reactions, including gluconeogenesis in the liver. This indicates that CA VA can act as a key mediator in several metabolic pathways of the liver, the only organ in which it is normally expressed [163,164,165]. Conversely, CA VB has broad tissue distribution even though it is the only CA isoform found in the mitochondria of adipocytes [166]. High expression of this isoform is also observed in the mitochondria of adrenal glands, tonsils, lymph nodes, spleen, liver, colon, and testis, and medium expression observed in other organs including the brain, lungs, muscles, GI tract, breast, and skin. CA VB functions within the adipocytes like CA VA: it stimulates pyruvate carboxylase activity, thereby increasing substrate levels for drawing off citrate for cytoplasmic transport [166,167]. Deficiencies resulting from alterations in CA5A, which decrease its enzymatic activity, causes hyperammonemia in early childhood [168]. 

### 4.2. Expression and Function of CA VA and CA VB in Tumor Cells

Although few studies have shown expression of CA VA and CA VB in cancer, the TCGA database shows high expression of CA5A in an RNA sequence study performed with liver hepatocellular carcinoma patient samples [141,142]. The highest gene alterations observed were amplifications, as seen in breast cancer patient xenografts (~17%) and pancreatic cancer (~6%), and deletions in prostate adenocarcinomas and metastatic cancer (~10%) (Figure 4B). No information is currently available for IHC detection of CA VA in tumors, either because none was observed or the studies are yet to be performed. CA5B mRNA upregulation in cancer is more widespread than CA5A with highest expression observed in acute myeloid leukemia, prostate, and renal cell carcinomas [141,142]. Once again, the most common alterations are amplification; in prostate and breast cancers, mutations were detected, including frameshift and missense mutations (Figure 2C). 

Most malignant cells exhibit moderate reactivity for CA VB in IHC staining [136,137,138,139]. A few cases of prostate and breast cancers showed strong staining. Most ovarian, skin, renal, urothelial, and gastric carcinomas were either weakly stained or negative. Because both isoforms are involved in metabolic processes, specifically lipogenesis, and tumors have been shown to upregulate lipogenesis, they could be important cancer targets. However, one might argue that since these isoforms are only found in the mitochondria of cells and mitochondrial dysfunction is one of the initiating factors of tumorigenesis, targeting mitochondrial CAs may not be as effective. It is also important to note that few studies have shown dysfunction in the Krebs cycle, specifically, as a result of mitochondrial dysregulation in the process of tumorigenesis. Citrate, an important precursor of fatty acid biosynthesis and lipogenesis, is an intermediate formed during this cycle in the mitochondria. While inhibition of the mitochondrial CAs alone might be effective, combining these inhibitors with drugs that directly target glycolysis, lipogenesis, and/or pH regulation may prove more potent for cancer therapy. These ideas must, however, be explored in more comprehensive and conclusive studies.

## 5. Secreted CA Isoforms: Expression, Distribution, and Function

CA VI is the only secreted isoform among the human CA family members [14,169]. It is distinct in terms of primary sequence and the number of conserved residues among the α-CAs (Table 1). The lowest percent sequence identity of 24.4% observed was with CA VB and the highest of 39% was surprisingly observed with CA IX (Table 1). CA VI has moderate catalytic activity and it is mostly expressed in salivary and mammary glands (Table 2). The physiological role of CA VI is still unclear, although it has been suggested that it is required for pH homeostasis of the mouth and taste perception [170,171,172]. 

### Expression and Function of CA VI in Normal and Tumor Cells

As the only secreted isoform, CA VI is also known as gustin [173]. It has been found in tears, milk, respiratory airways, human serum, epithelial lining of the alimentary canal, enamel organs, and most significantly in human saliva [174,175,176,177]. Although the physiological role of CA VI has not been established, it may be required for oral homeostasis to regulate against acidic environments [170]. Maintenance of proper pH levels in the saliva protects against enamel erosion and acid neutralization in biofilms and prevents dental caries [171,178,179,180]. Inhibition of CA VI was shown to cause taste perversion and sometimes complete loss of taste [173,181]. Exposure to high levels of exogenous zinc reversed the observed phenotype [173,181,182]. These studies suggest that CA VI plays a key role in taste perception and in maintaining proper salivary functions by regulating pH [181]. CA VI expression in cancer is limited both at mRNA and protein levels [136,141,142]. The most common gene alterations observed were missense mutations in melanoma followed by both deletions and amplifications (Figure 4D). No studies to date have linked CA VI to tumorigenesis, cancer progression, or metastasis. 

## 6. Membrane Associated CA Isoforms: Expression, Distribution, and Function

The membrane-associated CAs include the transmembrane isoforms: CA IX, XII, and XIV, and GPI-anchored isoform IV [3,4,48]. As might be expected, CA IV has the lowest sequence identity compared to the other membrane-associated isoforms, with an average of ~30% (Table 1). Surprisingly, CA XII and XIV are the most similar in terms of primary sequence and number of conserved residues, closely followed by CA IX and XIV. CA IX and XII have only 101 conserved residues, which equates to ~39% in primary sequence identity (Table 1). CA IV and CA IX are considered the fastest membrane-associated isoforms, with identical K_cat_ values of 1.1 × 10^6^ s^−1^, similar to that of CA II (Table 2) [183]. The expression and roles of the membrane-associated isoforms also varies from the kidneys, where they are necessary for bicarbonate reabsorption and normal kidney function, to the lungs, prostate, ovaries, GI tract, breast, and brain. They are also involved in hyperactivity of the heart and pH regulation/balance in retina, muscles, and erythrocytes [3,33,51,137]. More recently, CA IV and CA IX expression have been observed in cells that support wound repair [184]. Further, Membrane isoforms IX and XII have been implicated in tumorigenesis, cancer progression, and metastasis [36,37,38,41,110].

### 6.1. Expression and Function of CA IV and CA XIV in Normal Cells

High expression of GPI-anchored CA IV has been observed in the bone marrow, GI tract, liver, and gallbladder, whereas low expression is observed in the pancreas, kidney, brain, adipose, and soft tissues [17,185,186,187,188,189,190]. In the kidney, its function is necessary for bicarbonate reabsorption and normal kidney function [191]. Like CA II, CA IV has been shown to interact with various transporters [167,192]. These interactions increase the activity of bicarbonate transport and are required for maintaining appropriate pH balance within the environment of the retina and retinal pigment epithelium [193,194]. However, its mutant forms have been shown to be responsible for an autosomal dominant form of retinitis pigmentosa causing rod and cone photoreceptor degeneration [193,194]. CA IV has also been detected in tissue regeneration using mouse skin wound models [184]. These studies performed by Barker et al. showed increased CA IV mRNA during the period of wound hypoxia in keratinocytes, which form structures beneath the migrating epidermis [184]. In this setting, CA IV is suggested to contribute to wound healing by providing an acidic environment in which the migrating dermis and neutrophils can survive [184]. CA XIV mRNA shows strong expression in the brain, muscles, seminal vesicles, and retina [195,196]. IHC staining showed medium expression in different parts of the brain, muscles, and skin. Low CA XIV expression was observed in the liver, GI tract, seminal vesicles, and cervix [137,138,140]. Strong luminal correlation between CA IV and CA XIV suggest functional overlap between the enzymes [196]. CA XIV has also been shown to interact with bicarbonate transporters and has been implicated in acid–base balance in muscles and erythrocytes in response to chronic hypoxia, hyperactivity of the heart, and pH regulation in the retina [194,197,198].

### 6.2. Expression and Function of CA IV and CA XIV in Tumor Cells

**Figure 5:**
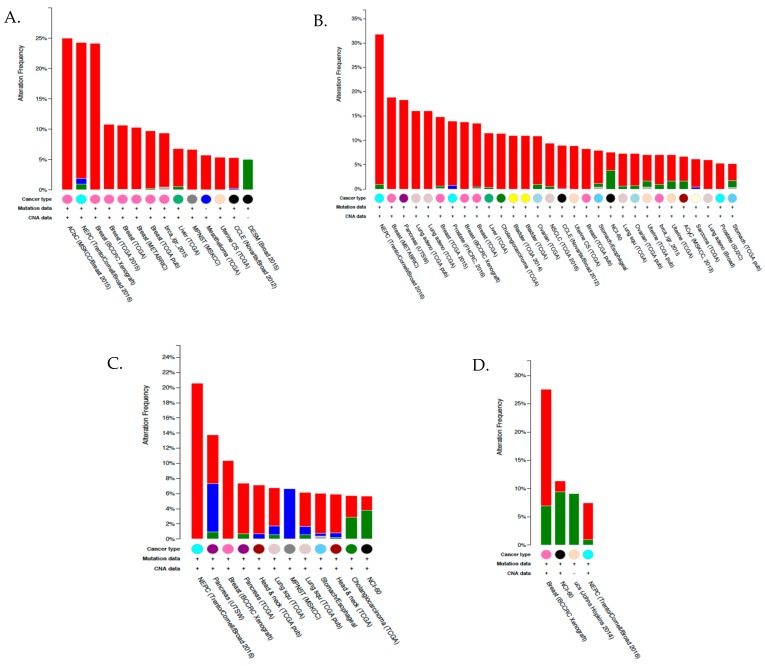
Summary of cross-cancer alterations for CA isoforms. (**A**) GPI-anchored CA IV, (**B**–**D**) transmembrane isoforms CA XIV, CA IX, and CA XIV, respectively. Gene amplifications are shown in red, mutations in green, deletions in blue, and multiple alterations in gray. The minimum percent altered samples threshold was set to 5%. The data and results were obtained from the cBioPortal of TCGA. Experimental details of each study referenced can also be found in the TCGA database.

The tumor-associated potential of isoforms CA IV and CA XIV, like many other α-CAs, has not been extensively studied. CA XIV mRNA has been shown to be upregulated in many cancers, being most often observed in melanomas, gliomas, liver, and uterine cancers [139,141,142]. It is the most altered gene in cancer among all the membrane-associated isoforms, with about >5% alterations, most of which are amplifications, based on 30 independent studies deposited in the TCGA (Figure 5B). CA XIV is seen in prostate, breast, pancreatic, lung, liver, bladder, and ovarian cancers. CA IV mRNA expression in cancer is much lower than CA XIV, but nonetheless can be observed in gliomas, renal cell carcinomas, thyroid cancers, and melanomas [139,141,142]. The most common gene alteration observed was amplification and the most common mutation was missense mutations (Figure 5A). However, CA IV IHC staining showed that cancer tissues were essentially negative. IHC detection of CA XIV showed moderate membranous staining in the majority of melanomas, along with a few hepatocellular carcinomas and pancreatic cancers [136,140]. Further studies specifically linking CA XIV to tumorigenesis and progression of the aforementioned and other cancers are yet to be explored and/or published.

### 6.3. Expression and Function of CA IX and CA XII in Normal Cells

CA IX was originally discovered by Pastorekova et al. as a component of MaTu quasi-viral agent [20]. Two years later, it was cloned and characterized as a tumor-associated member of the carbonic anhydrase family [199]. We now know that CA IX expression in normal adult tissue is limited to the outer shield of hair follicles, the epidermis of the skin during wound healing, and the GI tract [184,200]. In the GI tract CA IX is specifically found in the stomach and epithelial tissues of the gut, particularly the basolateral surfaces of the crypt, enterocytes of the duodenum, jejunum, and ileum of the small intestine [200]. In the large intestine, CA IX expression is restricted to the base of the glands in the cecum and colon [200,201]. Its function at these sites includes carbon dioxide and bicarbonate transport, acid–base balance, and signal transduction. Because of the presence of an “exofacial” proteoglycan-like domain in CA IX, which functions independently of its catalytic activity, it is also thought to contribute to the assembly and maturation of focal adhesion contacts during initial cell spreading [50,56,202,203]. In contrast, expression of CA XII has been observed in many normal tissues with high expression reported in the appendix, pancreas, colon, rectum, kidney, prostate, intestine, and activated lymphocytes. Moderate expression is observed in the esophagus, oral mucosa, urinary bladder, breast, vagina, cervix, endometrium, and skin, and low expression is seen in the stomach, seminal vesicles, and fallopian tubes [38,120,200,204,205]. Its biological functions in normal tissue range from facilitating bicarbonate transport in cells to maintenance of an internal steady state concentration of chloride ions within an organism [206,207]. Similar to CA IX, CA XII is also involved in chemical reactions and pathways involving small molecule metabolism. Furthermore, it has been postulated that CA XII may be important for normal kidney function [191,205].

### 6.4. Expression and Function of CA IX and CA XII in Tumor Cells

Both CA IX and CA XII are often regarded as tumor-associated CAs and thus are the most studied in the tumor setting. As we have discussed in previous sections, both CA IX and XII act in conjunction with cyt-CAs to establish the differential pH in the cellular microenvironment observed in hypoxic tumors [56,110,167]. CA IX has, however, garnered more attention because its limited expression in normal cells and upregulation in many aggressive cancers compared to CA XII. This suggests that CA IX is a more “druggable” target. Cancers associated with CA IX expression include brain, breast, bladder, cervix, colon, colorectal, head and neck, pancreas, kidney, lung, ovaries, stomach oral cavity, and T-cell lymphomas [52,68,136,139,208,209,210]. CA IX expression, especially in hypoxic tumors, is modulated by hypoxia-inducible factor 1 (HIF-1) in response to low oxygen levels and increased cell density [211]. CA IX expression can also be regulated both in chronic and mild hypoxic conditions by components of the mitogen-activated protein kinase (MAPK) pathway [212,213]. Studies have demonstrated that the MAPK pathway controls the CA9 promoter, using both the HIF-1-dependent and -independent signals, working as a downstream mediator of CA9 transcriptional response to both hypoxia and high cell density [213]. This is important since activating mutations of the MAPK and phosphatidylinositol-3 kinase (PI3K) pathways, which occur in many tumor types, may consequently upregulate CA9 gene expression and influence intratumoral distribution of the CA IX protein. Furthermore, there is evidence of CA IX expression at the leading edge of cells, displaying a migratory phenotype where it may serve as a modulator of tumor aggression [214].

Regulation of CA IX activity has been observed through phosphorylation of its intracellular domain at three putative sites. Phosphorylation at Thr 443 by protein kinase A (PKA) in response to cyclic adenosine monophosphate (cAMP) activates CA IX activity, under hypoxic conditions [215]. Epidermal growth factor (EGF) induced phosphorylation at the same position mediates cross talk between CA IX and PI3K to activate Akt kinase, although the impact on CA IX activity is unclear [216]. On the other hand, phosphorylation of CA IX at Ser 448 inhibits enzyme activity. The signal transduction activity of CA IX is activated when the tyrosine residue at position 449 is phosphorylated [215,216]. CA IX has also been shown to be a key modulator of tumor growth, survival and migration, tumorigenesis, pH control, cell adhesion, and proliferation, and is the basis of studies used to establish mechanisms proposed in both Hypothesis 1 and 2 presented in earlier sections (Figure 1) [33,52,202,217,218]. CA IX expression is regulated by tumor hypoxia and has not only been established as a prognostic indicator for a variety of cancers but also as an anticancer target. Overexpression of CA IX in most cancers is associated with poor prognosis, chemotherapeutic resistance, and poor clinical outcome [219,220,221,222]. 

Like CA IX, CA XII expression has also been shown to be upregulated in many tumor types. However, its expression is also abundant in normal tissues. The most abundant cancer-related CA12 expression, according to RNA sequencing datasets deposited in the TCGA database, was observed in renal cell carcinomas, colorectal, breast, bladder glioblastomas, and head and neck cancers [141,142]. The most common gene alterations include amplifications and mutations in breast cancer patient xenografts, mutations in NCI-60 cell lines, and uterine carcinomas (Figure 5D). Several cases of breast, renal, urothelial, skin, lung, endometrial, and cervical cancers also displayed moderate to strong membranous IHC staining, with additional positivity in a few cases [136,139]. Furthermore, high expression of CA XII has also been observed in glioblastomas, astrocytomas, and T-cell lymphomas [136]. Despite all the evidence that suggested CA XII protein has potential as a prognosticator, it has still not been fully established as a prognostic marker. This may be due to the fact that in some cancers it is a marker of “good prognosis,” including in lung, cervical, and breast cancer, whereas its expression has no prognostic value in other types such as brain cancer [40,223,224,225]. Finally, in cancers such as colorectal, oral squamous carcinoma, and some kidney cancers, CA XII upregulation is associated with poor prognosis [205,226]. Recent studies have shown increased CA XII expression on the surface of chemoresistant cells, suggesting its potential as a therapeutic target to overcome chemoresistance in cancer cells [227]. Furthermore, CA XII has been shown to facilitate cancer cell survival and promote tumor cell migration, invasion, and maintenance of cancer cell stemness [36,53,205].

## 7. Conclusions

One current research interest in the field of CA drug discovery is enzymes’ role in cancer, either as a prognostic marker or as a potential drug target. To date, experimental evidence has indicated an important relationship between pH regulation and tumor cell proliferation and survival. Many of these studies show direct involvement of CA in these processes, marking them as important avenues for pH-disruptive anti-cancer treatments. This is particular true in tumors that have undergone hypoxic transition, where CAs establish pH gradients that favor cancer cell growth at the expense of adjacent normal cell (and patient) survival. Of the 15 human CAs, however, attention has only been directed toward two isoforms, CA IX and XII. Of course, this has been fruitful as inhibitors are now in clinical trials that specifically target each isoform. Despite this achievement, the roles of the remaining CAs have only recently been considered in cancers. This area of research has strong potential as many of the human isoforms, beyond CA IX and XII, have shown an association with tumorigenesis in primary cancers. With much still to understand in terms of the “combined role” of CAs in different cancer types, it will be important moving forward for researchers to consider the effects beside those of CA IX and XII. As the search for viable and novel treatments against the most aggressive and detrimental cancers continues, priority should be placed on deciphering the roles of cancer-related CAs. 

## Figures and Tables

**Table 1:** Primary sequence identity (%) (italicized, bottom left) and number of conserved residues (right, top) between the catalytically active CA isoforms.

	I	II	III	IV	VA	VB	VI	VII	IX	XII	XIII	XIV
**I**	-	154	141	78	126	128	82	132	83	91	154	85
**II**	*60*	-	152	88	133	138	90	147	85	89	157	96
**III**	*54*	*58*	-	82	120	117	87	130	80	86	151	90
**IV**	*30*	*33*	*32*	-	89	93	97	90	84	91	84	62
**VA**	*48*	*51*	*45*	*24*	-	184	93	131	83	84	124	88
**VB**	*47*	*52*	*43*	*23*	*59*	-	82	134	89	79	131	88
**VI**	*32*	*33*	*32*	*27*	*28*	*24*	-	93	107	104	90	106
**VII**	*51*	*56*	*50*	*32*	*48*	*49*	*35*	-	95	103	139	97
**IX**	*33*	*34*	*31*	*27*	*32*	*33*	*39*	*37*	-	101	90	113
**XII**	*36*	*34*	*32*	*28*	*32*	*30*	*38*	*33*	*39*	-	91	123
**XIII**	*59*	*60*	*58*	*28*	*46*	*48*	*33*	*53*	*35*	*35*	-	98
**XIV**	*34*	*36*	*34*	*29*	*32*	*29*	*36*	*36*	*44*	*46*	*37*	-

Information adapted from Pinard et al. [48].

**Table 2:** Catalytic efficiency of the CA isoforms.

Isoform	K_cat_ (s^–1^)	K_M_ (mM)	K_cat_/K_M_ (M^−1^ s^−1^)
**I**	2.0 × 10^5^	4.0	5.0 × 10^7^
**II**	1.4 × 10^6^	9.3	1.5 × 10^8^
**III**	1.3 × 10^4^	33.3	4.0 × 10^5^
**IV**	1.1 × 10^6^	21.5	5.1 × 10^7^
**VA**	2.9 × 10^5^	10.0	2.9 × 10^7^
**VB**	9.5 × 10^5^	9.7	9.8 × 10^7^
**VI**	3.4 × 10^5^	6.9	4.9 × 10^7^
**VII**	9.5 × 10^6^	11.4	8.3 × 10^7^
**IX**	1.1 × 10^6^	6.9	1.6 × 10^8^
**XII**	4.2 × 10^5^	12.0	3.5 × 10^7^
**XIII**	1.5 × 10^5^	13.8	1.1 × 10^7^
**XIV**	3.1 × 10^5^	7.9	3.9 × 10^7^

## References

[1] Stadie W.C., O’Brien H. (1933). The catalytic hydration of carbondioxide and dehydration of carbonic acid by enzyme isolated from red blood cells. J. Biol. Chem..

[2] Supuran C.T. (2016). Structure and function of carbonic anhydrases. Biochem. J..

[3] Frost S.C., McKenna R. (2014). Carbonic Anhydrase: Mechanism, Regulation, Links to Disease, and Industrial Applications, Subcellular Biochemistry.

[4] Supuran C.T. (2008). Carbonic anhydrases—An overview. Curr. Pharm. Des..

[5] Del Prete S., Vullo D., Fisher G.M., Andrews K.T., Poulsen S.-A., Capasso C., Supuran C.T. (2014). Discovery of a new family of carbonic anhydrases in the malaria pathogen *Plasmodium falciparum*—the η-carbonic anhydrases. Bioorg. Med. Chem. Lett..

[6] Nyman P., Lindskog S. (1964). Amino acid composition of various forms of bovine and human erythrocyte carbonic anhydrase. Biochim. Biophys. Acta.

[7] Andersson B., Nyman P.O., Strid L. (1972). Amino acid sequence of human erythrocyte carbonic anhydrase B. Biochem. Biophys. Res. Commun..

[8] Lindskog S. (1960). Purification and properties of bovine erythrocyte carbonic anhydrase. Biochim. Biophys. Acta.

[9] Nyman P.O. (1961). Purification and properties of carbonic anhydrase from human erythrocytes. Biochim. Biophys. Acta.

[10] Liljas A., Kannan K.K., Bergstén P.C., Waara I., Fridborg K., Strandberg B., Carlbom U., Järup L., Lövgren S., Petef M. (1972). Crystal structure of human carbonic anhydrase C. Nat. New Biol..

[11] Lin K.T., Deutsch H.F. (1973). Human carbonic anhydrases. XI. The complete primary structure of carbonic anhydrase B. J. Biol. Chem..

[12] Balckburn M.N., Chirgwin J.M., Gordon J.T., Thomas K.D., Parsons T.F., Register A.M., Schnackerz K.D., Noltmann E.A. (1972). Pseudoisoenzymes of rabbit muscle phosphoglucose isomerase. J. Biol. Chem..

[13] Fernley R.T., Wright R.D., Coghlan J.P. (1979). A novel carbonic anhydrase from the ovine parotid gland. FEBS Lett..

[14] Murakami H., Sly W.S. (1987). Purification and characterization of human salivary carbonic anhydrase. J. Biol. Chem..

[15] Whitney P.L., Briggle T.V. (1982). Membrane-associated carbonic anhydrase purified from bovine lung. J. Biol. Chem..

[16] Wistrand P.J. (1984). Properties of membrane-bound carbonic anhydrase. Ann. N. Y. Acad. Sci..

[17] Carter N.D., Fryer A., Grant A.G., Hume R., Strange R.G., Wistrand P.J. (1990). Membrane specific carbonic anhydrase (CAIV) expression in human tissues. Biochim. Biophys. Acta.

[18] Zhu X.L., Sly W.S. (1990). Carbonic anhydrase IV from human lung. Purification, characterization, and comparison with membrane carbonic anhydrase from human kidney. J. Biol. Chem..

[19] Montgomery J.C., Venta P.J., Eddy R.L., Fukushima Y.-S., Shows T.B., Tashian R.E. (1991). Characterization of the human gene for a newly discovered carbonic anhydrase, CA VII, and its localization to chromosome 16. Genomics.

[20] Pastoreková S., Závadová Z., Kostál M., Babusíková O., Závada J. (1992). A novel quasi-viral agent, MaTu, is a two-component system. Virology.

[21] Opavský R., Pastoreková S., Zelník V., Gibadulinová A., Stanbridge E.J., Závada J., Kettmann R., Pastorek J. (1996). Human MN/CA9 gene, a novel member of the carbonic anhydrase family: Structure and exon to protein domain relationships. Genomics.

[22] Türeci O., Sahin U., Vollmar E., Siemer S., Göttert E., Seitz G., Parkkila A.K., Shah G.N., Grubb J.H., Pfreundschuh M. (1998). Human carbonic anhydrase XII: CDNA cloning, expression, and chromosomal localization of a carbonic anhydrase gene that is overexpressed in some renal cell cancers. Proc. Natl. Acad. Sci. USA.

[23] Fujikawa-Adachi K., Nishimori I., Taguchi T., Onishi S. (1999). Human carbonic anhydrase XIV (CA14): CDNA cloning, mRNA expression, and mapping to chromosome 1. Genomics.

[24] Mori K., Ogawa Y., Ebihara K., Tamura N., Tashiro K., Kuwahara T., Mukoyama M., Sugawara A., Ozaki S., Tanaka I. (1999). Isolation and characterization of CA XIV, a novel membrane-bound carbonic anhydrase from mouse kidney. J. Biol. Chem..

[25] Nagao Y., Platero J.S., Waheed A., Sly W.S. (1993). Human mitochondrial carbonic anhydrase: CDNA cloning, expression, subcellular localization, and mapping to chromosome 16. Proc. Natl. Acad. Sci. USA.

[26] Fujikawa-Adachi K., Nishimori I., Taguchi T., Onishi S. (1999). Human mitochondrial carbonic anhydrase VB: CDNA cloning, mRNA expression, subcellular localization, and mapping to chromosome X. J. Biol. Chem..

[27] Idrees D., Kumar S., Rehman S.A.A., Gourinath S., Islam A., Ahmad F., Imtaiyaz Hassan M. (2016). Cloning, expression, purification and characterization of human mitochondrial carbonic anhydrase VA. 3 Biotech.

[28] Lehtonen J., Shen B., Vihinen M., Casini A., Scozzafava A., Supuran C.T., Parkkila A.-K., Saarnio J., Kivelä A.J., Waheed A. (2004). Characterization of CA XIII, a novel member of the carbonic anhydrase isozyme family. J. Biol. Chem..

[29] Ozensoy Guler O., Capasso C., Supuran C.T. (2015). A magnificent enzyme superfamily: Carbonic anhydrases, their purification and characterization. J. Enzyme Inhib. Med. Chem..

[30] Supuran C.T. (2008). Carbonic anhydrases: Novel therapeutic applications for inhibitors and activators. Nat. Rev. Drug Discov..

[31] Thiry A., Dogné J.-M., Supuran C.T., Masereel B. (2007). Carbonic anhydrase inhibitors as anticonvulsant agents. Curr. Top. Med. Chem..

[32] Supuran C.T. (2016). How many carbonic anhydrase inhibition mechanisms exist?. J. Enzyme Inhib. Med. Chem..

[33] Li Y., Tu C., Wang H., Silverman D.N., Frost S.C. (2011). Catalysis and pH control by membrane-associated carbonic anhydrase IX in MDA-MB-231 breast cancer cells. J. Biol. Chem..

[34] Sly W.S. (2000). The membrane carbonic anhydrases: From CO_2_ transport to tumor markers. EXS.

[35] Nordfors K., Haapasalo J., Korja M., Niemelä A., Laine J., Parkkila A.-K., Pastorekova S., Pastorek J., Waheed A., Sly W.S. (2010). The tumour-associated carbonic anhydrases CA II, CA IX and CA XII in a group of medulloblastomas and supratentorial primitive neuroectodermal tumours: An association of CA IX with poor prognosis. BMC Cancer.

[36] Lounnas N., Rosilio C., Nebout M., Mary D., Griessinger E., Neffati Z., Chiche J., Spits H., Hagenbeek T.J., Asnafi V. (2013). Pharmacological inhibition of carbonic anhydrase XII interferes with cell proliferation and induces cell apoptosis in T-cell lymphomas. Cancer Lett..

[37] Saarnio J., Parkkila S., Parkkila A.K., Haukipuro K., Pastoreková S., Pastorek J., Kairaluoma M.I., Karttunen T.J. (1998). Immunohistochemical study of colorectal tumors for expression of a novel transmembrane carbonic anhydrase, MN/CA IX, with potential value as a marker of cell proliferation. Am. J. Pathol..

[38] Kivelä A.J., Parkkila S., Saarnio J., Karttunen T.J., Kivelä J., Parkkila A.K., Pastoreková S., Pastorek J., Waheed A., Sly W.S. (2000). Expression of transmembrane carbonic anhydrase isoenzymes IX and XII in normal human pancreas and pancreatic tumours. Histochem. Cell Biol..

[39] Kummola L., Hämäläinen J.M., Kivelä J., Kivelä A.J., Saarnio J., Karttunen T., Parkkila S. (2005). Expression of a novel carbonic anhydrase, CA XIII, in normal and neoplastic colorectal mucosa. BMC Cancer.

[40] Barnett D.H., Sheng S., Charn T.H., Waheed A., Sly W.S., Lin C.-Y., Liu E.T., Katzenellenbogen B.S. (2008). Estrogen receptor regulation of carbonic anhydrase XII through a distal enhancer in breast cancer. Cancer Res..

[41] Chen L.Q., Howison C.M., Spier C., Stopeck A.T., Malm S.W., Pagel M.D., Baker A.F. (2015). Assessment of carbonic anhydrase IX expression and extracellular pH in B-cell lymphoma cell line models. Leuk. Lymphoma.

[42] Savile C.K., Lalonde J.J. (2011). Biotechnology for the acceleration of carbon dioxide capture and sequestration. Curr. Opin. Biotechnol..

[43] Bond G.M., Stringer J., Brandvold D.K., Simsek F.A., Medina M.-G., Egeland G. (2001). Development of integrated system for biomimetic CO_2_ sequestration using the enzyme carbonic anhydrase. Energy Fuels.

[44] Lee S.-W., Park S.-B., Jeong S.-K., Lim K.-S., Lee S.-H., Trachtenberg M.C. (2010). On carbon dioxide storage based on biomineralization strategies. Micron.

[45] Kaar J.L., Oh H.-I., Russell A.J., Federspiel W.J. (2007). Towards improved artificial lungs through bio-catalysis. Biomaterials.

[46] Sreenivasan R., Bassett E.K., Hoganson D.M., Vacanti J.P., Gleason K.K. (2011). Ultra-thin, gas permeable free-standing and composite membranes for microfluidic lung assist devices. Biomaterials.

[47] Stadermann M., Baxamusa S.H., Aracne-Ruddle C., Chea M., Li S., Youngblood K., Suratwala T. (2015). Fabrication of Large-area Free-standing Ultrathin Polymer Films. J. Vis. Exp..

[48] Pinard M.A., Mahon B., McKenna R. (2015). Probing the surface of human carbonic anhydrase for clues towards the design of isoform specific inhibitors. BioMed Res. Int..

[49] Mboge M.Y., McKenna R., Frost S.C. (2016). Advances in anti-cancer drug development targeting carbonic anhydrase IX and XII. Topics in Anti-Cancer Research.

[50] Mahon B.P., Pinard M.A., McKenna R. (2015). Targeting carbonic anhydrase IX activity and expression. Molecules.

[51] Mahon B.P., Bhatt A., Socorro L., Driscoll J.M., Okoh C., Lomelino C.L., Mboge M.Y., Kurian J.J., Tu C., Agbandje-McKenna M. (2016). The structure of carbonic anhydrase IX is adapted for low-pH catalysis. Biochemistry.

[52] Yang J.-S., Lin C.-W., Chuang C.-Y., Su S.-C., Lin S.-H., Yang S.-F. (2015). Carbonic anhydrase IX overexpression regulates the migration and progression in oral squamous cell carcinoma. Tumour Biol. J. Int. Soc. Oncodev. Biol. Med..

[53] Hsieh M.-J., Chen K.-S., Chiou H.-L., Hsieh Y.-S. (2010). Carbonic anhydrase XII promotes invasion and migration ability of MDA-MB-231 breast cancer cells through the p38 MAPK signaling pathway. Eur. J. Cell Biol..

[54] Pacchiano F., Carta F., McDonald P.C., Lou Y., Vullo D., Scozzafava A., Dedhar S., Supuran C.T. (2011). Ureido-substituted benzenesulfonamides potently inhibit carbonic anhydrase IX and show antimetastatic activity in a model of breast cancer metastasis. J. Med. Chem..

[55] Winum J.-Y., Carta F., Ward C., Mullen P., Harrison D., Langdon S.P., Cecchi A., Scozzafava A., Kunkler I., Supuran C.T. (2012). Ureido-substituted sulfamates show potent carbonic anhydrase IX inhibitory and antiproliferative activities against breast cancer cell lines. Bioorg. Med. Chem. Lett..

[56] Mahon B.P., McKenna R. (2013). Regulation and role of carbonic anhydrase IX and use as a biomarker and therapeutic target in cancer. Res. Trends Curr. Top. Biochem. Res..

[57] Supuran C.T. (2017). Carbonic Anhydrase inhibition and the management of hypoxic tumors. Metabolites.

[58] Mulders P., Bleumer I., Debruyne F., Oosterwijk E. (2004). Specific monoclonal antibody-based immunotherapy by targeting the RCC-associated antigen carbonic anhydrase-IX(G250/MN). Urol. Ausg A.

[59] Davis I.D., Wiseman G.A., Lee F.-T., Gansen D.N., Hopkins W., Papenfuss A.T., Liu Z., Moynihan T.J., Croghan G.A., Adjei A.A. (2007). A phase I multiple dose, dose escalation study of cG250 monoclonal antibody in patients with advanced renal cell carcinoma. Cancer Immun..

[60] Stillebroer A.B., Boerman O.C., Desar I.M.E., Boers-Sonderen M.J., van Herpen C.M.L., Langenhuijsen J.F., Smith-Jones P.M., Oosterwijk E., Oyen W.J.G., Mulders P.F.A. (2013). Phase 1 radioimmunotherapy study with lutetium 177-labeled anti-carbonic anhydrase IX monoclonal antibody girentuximab in patients with advanced renal cell carcinoma. Eur. Urol..

[61] Muselaers C.H.J., Boers-Sonderen M.J., van Oostenbrugge T.J., Boerman O.C., Desar I.M.E., Stillebroer A.B., Mulder S.F., van Herpen C.M.L., Langenhuijsen J.F., Oosterwijk E. (2016). Phase 2 study of lutetium 177-labeled anti-carbonic anhydrase IX monoclonal antibody girentuximab in patients with advanced renal cell carcinoma. Eur. Urol..

[62] Dubois L.J., Niemans R., van Kuijk S.J.A., Panth K.M., Parvathaneni N.-K., Peeters S.G.J.A., Zegers C.M.L., Rekers N.H., van Gisbergen M.W., Biemans R. (2015). New ways to image and target tumour hypoxia and its molecular responses. Radiother. Oncol..

[63] Li J., Zhang G., Wang X., Li X.-F. (2015). Is carbonic anhydrase IX a validated target for molecular imaging of cancer and hypoxia?. Future Oncol..

[64] Ahlskog J.K.J., Schliemann C., Mårlind J., Qureshi U., Ammar A., Pedley R.B., Neri D. (2009). Human monoclonal antibodies targeting carbonic anhydrase IX for the molecular imaging of hypoxic regions in solid tumours. Br. J. Cancer.

[65] Chen F., Zhuang X., Lin L., Yu P., Wang Y., Shi Y., Hu G., Sun Y. (2015). New horizons in tumor microenvironment biology: Challenges and opportunities. BMC Med..

[66] Whiteside T.L. (2008). The tumor microenvironment and its role in promoting tumor growth. Oncogene.

[67] Brown J.M. (2007). Tumor hypoxia in cancer therapy. Methods Enzymol..

[68] Jamali S., Klier M., Ames S., Felipe Barros L., McKenna R., Deitmer J.W., Becker H.M. (2015). Hypoxia-induced carbonic anhydrase IX facilitates lactate flux in human breast cancer cells by non-catalytic function. Sci. Rep..

[69] Warburg O. (1928). The chemical constitution of respiratory ferment. Science.

[70] Warburg O., Wind F., Negelein E. (1927). The metabolism of tumors in the body. J. Gen. Physiol..

[71] Gatenby R.A., Gillies R.J. (2004). Why do cancers have high aerobic glycolysis?. Nat. Rev. Cancer.

[72] Newsholme P., Lima M.M.R., Procopio J., Pithon-Curi T.C., Doi S.Q., Bazotte R.B., Curi R. (2003). Glutamine and glutamate as vital metabolites. Braz. J. Med. Biol. Res..

[73] Brekke E., Morken T.S., Walls A.B., Waagepetersen H., Schousboe A., Sonnewald U. (2016). Anaplerosis for glutamate synthesis in the neonate and in adulthood. The Glutamate/GABA-Glutamine Cycle.

[74] DeBerardinis R.J., Mancuso A., Daikhin E., Nissim I., Yudkoff M., Wehrli S., Thompson C.B. (2007). Beyond aerobic glycolysis: Transformed cells can engage in glutamine metabolism that exceeds the requirement for protein and nucleotide synthesis. Proc. Natl. Acad. Sci. USA.

[75] Currie E., Schulze A., Zechner R., Walther T.C., Farese R.V. (2013). Cellular fatty acid metabolism and cancer. Cell Metab..

[76] Pavlova N.N., Thompson C.B. (2016). The emerging hallmarks of cancer metabolism. Cell Metab..

[77] Gillies R.J., Raghunand N., Karczmar G.S., Bhujwalla Z.M. (2002). MRI of the tumor microenvironment. J. Magn. Reson. Imaging JMRI.

[78] Webb B.A., Chimenti M., Jacobson M.P., Barber D.L. (2011). Dysregulated pH: A perfect storm for cancer progression. Nat. Rev. Cancer.

[79] Stüwe L., Müller M., Fabian A., Waning J., Mally S., Noël J., Schwab A., Stock C. (2007). pH dependence of melanoma cell migration: Protons extruded by NHE1 dominate protons of the bulk solution. J. Physiol..

[80] Balkwill F.R., Capasso M., Hagemann T. (2012). The tumor microenvironment at a glance. J. Cell Sci..

[81] Wilson W.R., Hay M.P. (2011). Targeting hypoxia in cancer therapy. Nat. Rev. Cancer.

[82] Dietl K., Renner K., Dettmer K., Timischl B., Eberhart K., Dorn C., Hellerbrand C., Kastenberger M., Kunz-Schughart L.A., Oefner P.J. (2010). Lactic acid and acidification inhibit TNF secretion and glycolysis of human monocytes. J. Immunol..

[83] Kuwata F., Suzuki N., Otsuka K., Taguchi M., Sasai Y., Wakino H., Ito M., Ebihara S., Suzuki K. (1991). Enzymatic regulation of glycolysis and gluconeogenesis in rabbit periodontal ligament under various physiological pH conditions. J. Nihon Univ. Sch. Dent..

[84] Chan F.K.-M., Moriwaki K., De Rosa M.J. (2013). Detection of necrosis by release of lactate dehydrogenase activity. Immune Homeostasis.

[85] Gray J.A. (1966). Kinetics of enamel dissolution during formation of incipient caries-like lesions. Arch. Oral Biol..

[86] Putney L.K., Barber D.L. (2004). Expression profile of genes regulated by activity of the Na-H exchanger NHE1. BMC Genom..

[87] Chen J.L.-Y., Lucas J.E., Schroeder T., Mori S., Wu J., Nevins J., Dewhirst M., West M., Chi J.-T. (2008). The genomic analysis of lactic acidosis and acidosis response in human cancers. PLoS Genet..

[88] Menard L., Maughan D., Vigoreaux J. (2014). The structural and functional coordination of glycolytic enzymes in muscle: Evidence of a metabolon?. Biology.

[89] Campanella M.E., Chu H., Wandersee N.J., Peters L.L., Mohandas N., Gilligan D.M., Low P.S. (2008). Characterization of glycolytic enzyme interactions with murine erythrocyte membranes in wild-type and membrane protein knockout mice. Blood.

[90] Stock C., Schwab A. (2009). Protons make tumor cells move like clockwork. Pflugers Arch..

[91] Wilhelm S.M., Shao Z.H., Housley T.J., Seperack P.K., Baumann A.P., Gunja-Smith Z., Woessner J.F. (1993). Matrix metalloproteinase-3 (stromelysin-1). Identification as the cartilage acid metalloprotease and effect of pH on catalytic properties and calcium affinity. J. Biol. Chem..

[92] Bourguignon L.Y.W., Singleton P.A., Diedrich F., Stern R., Gilad E. (2004). CD44 interaction with Na^+^-H^+^ exchanger (NHE1) creates acidic microenvironments leading to hyaluronidase-2 and cathepsin B activation and breast tumor cell invasion. J. Biol. Chem..

[93] Lee H.-S., Bellin R.M., Walker D.L., Patel B., Powers P., Liu H., Garcia-Alvarez B., de Pereda J.M., Liddington R.C., Volkmann N. (2004). Characterization of an actin-binding site within the talin FERM domain. J. Mol. Biol..

[94] Moseley J.B., Okada K., Balcer H.I., Kovar D.R., Pollard T.D., Goode B.L. (2006). Twinfilin is an actin-filament-severing protein and promotes rapid turnover of actin structures in vivo. J. Cell Sci..

[95] Pope B.J., Zierler-Gould K.M., Kühne R., Weeds A.G., Ball L.J. (2004). Solution structure of human cofilin: Actin binding, pH sensitivity, and relationship to actin-depolymerizing factor. J. Biol. Chem..

[96] Srivastava J., Barreiro G., Groscurth S., Gingras A.R., Goult B.T., Critchley D.R., Kelly M.J.S., Jacobson M.P., Barber D.L. (2008). Structural model and functional significance of pH-dependent talin-actin binding for focal adhesion remodeling. Proc. Natl. Acad. Sci. USA.

[97] Grey M.J., Tang Y., Alexov E., McKnight C.J., Raleigh D.P., Palmer A.G. (2006). Characterizing a partially folded intermediate of the villin headpiece domain under non-denaturing conditions: Contribution of His41 to the pH-dependent stability of the N-terminal subdomain. J. Mol. Biol..

[98] McLachlan G.D., Cahill S.M., Girvin M.E., Almo S.C. (2007). Acid-induced equilibrium folding intermediate of human platelet profilin. Biochemistry.

[99] Lagadic-Gossmann D., Huc L., Lecureur V. (2004). Alterations of intracellular pH homeostasis in apoptosis: Origins and roles. Cell Death Differ..

[100] Matsuyama S., Llopis J., Deveraux Q.L., Tsien R.Y., Reed J.C. (2000). Changes in intramitochondrial and cytosolic pH: Early events that modulate caspase activation during apoptosis. Nat. Cell Biol..

[101] Pouysségur J., Franchi A., L’Allemain G., Paris S. (1985). Cytoplasmic pH, a key determinant of growth factor-induced DNA synthesis in quiescent fibroblasts. FEBS Lett..

[102] Bower J.J., Vance L.D., Psioda M., Smith-Roe S.L., Simpson D.A., Ibrahim J.G., Hoadley K.A., Perou C.M., Kaufmann W.K. (2017). Patterns of cell cycle checkpoint deregulation associated with intrinsic molecular subtypes of human breast cancer cells. Npj Breast Cancer.

[103] Khaled A.R., Kim K., Hofmeister R., Muegge K., Durum S.K. (1999). Withdrawal of IL-7 induces Bax translocation from cytosol to mitochondria through a rise in intracellular pH. Proc. Natl. Acad. Sci. USA.

[104] Swietach P., Vaughan-Jones R.D., Harris A.L., Hulikova A. (2014). The chemistry, physiology and pathology of pH in cancer. Philos. Trans. R. Soc. B Biol. Sci..

[105] Neri D., Supuran C.T. (2011). Interfering with pH regulation in tumours as a therapeutic strategy. Nat. Rev. Drug Discov..

[106] Uda N.R., Seibert V., Stenner-Liewen F., Müller P., Herzig P., Gondi G., Zeidler R., van Dijk M., Zippelius A., Renner C. (2015). Esterase activity of carbonic anhydrases serves as surrogate for selecting antibodies blocking hydratase activity. J. Enzyme Inhib. Med. Chem..

[107] Verpoorte J.A., Mehta S., Edsall J.T. (1967). Esterase activities of human carbonic anhydrases B and C. J. Biol. Chem..

[108] Lindskog S., Coleman J.E. (1973). The catalytic mechanism of carbonic anhydrase. Proc. Natl. Acad. Sci. USA.

[109] Al-Samir S., Papadopoulos S., Scheibe R.J., Meißner J.D., Cartron J.-P., Sly W.S., Alper S.L., Gros G., Endeward V. (2013). Activity and distribution of intracellular carbonic anhydrase II and their effects on the transport activity of anion exchanger AE1/SLC4A1: Role of CAII in the function of AE1. J. Physiol..

[110] Benej M., Pastorekova S., Pastorek J. (2014). Carbonic anhydrase IX: Regulation and role in cancer. Subcell. Biochem..

[111] Alterio V., Hilvo M., Di Fiore A., Supuran C.T., Pan P., Parkkila S., Scaloni A., Pastorek J., Pastorekova S., Pedone C. (2009). Crystal structure of the catalytic domain of the tumor-associated human carbonic anhydrase IX. Proc. Natl. Acad. Sci. USA.

[112] Widmann M., Trodler P., Pleiss J. (2010). The isoelectric region of proteins: A systematic analysis. PLoS ONE.

[113] Klier M., Andes F.T., Deitmer J.W., Becker H.M. (2014). Intracellular and extracellular carbonic anhydrases cooperate non-enzymatically to enhance activity of monocarboxylate transporters. J. Biol. Chem..

[114] Becker H.M., Klier M., Schüler C., McKenna R., Deitmer J.W. (2011). Intramolecular proton shuttle supports not only catalytic but also noncatalytic function of carbonic anhydrase II. Proc. Natl. Acad. Sci. USA.

[115] Svastova E., Witarski W., Csaderova L., Kosik I., Skvarkova L., Hulikova A., Zatovicova M., Barathova M., Kopacek J., Pastorek J. (2012). Carbonic anhydrase IX interacts with bicarbonate transporters in lamellipodia and increases cell migration via its catalytic domain. J. Biol. Chem..

[116] Innocenti A., Vullo D., Scozzafava A., Casey J.R., Supuran C.T. (2005). Carbonic anhydrase inhibitors. Interaction of isozymes I, II, IV, V, and IX with carboxylates. Bioorg. Med. Chem. Lett..

[117] Swietach P., Patiar S., Supuran C.T., Harris A.L., Vaughan-Jones R.D. (2009). The role of carbonic anhydrase 9 in regulating extracellular and intracellular pH in three-dimensional tumor cell growths. J. Biol. Chem..

[118] Giffard R.G., Monyer H., Christine C.W., Choi D.W. (1990). Acidosis reduces NMDA receptor activation, glutamate neurotoxicity, and oxygen-glucose deprivation neuronal injury in cortical cultures. Brain Res..

[119] The PyMOL Molecular Graphics System, Version 2.0 Schrödinger, LLC. https://pymol.org/2/.

[120] Whittington D.A., Waheed A., Ulmasov B., Shah G.N., Grubb J.H., Sly W.S., Christianson D.W. (2001). Crystal structure of the dimeric extracellular domain of human carbonic anhydrase XII, a bitopic membrane protein overexpressed in certain cancer tumor cells. Proc. Natl. Acad. Sci. USA.

[121] Zhao J., Benlekbir S., Rubinstein J.L. (2015). Electron cryomicroscopy observation of rotational states in a eukaryotic V-ATPase. Nature.

[122] Arakawa T., Kobayashi-Yurugi T., Alguel Y., Iwanari H., Hatae H., Iwata M., Abe Y., Hino T., Ikeda-Suno C., Kuma H. (2015). Crystal structure of the anion exchanger domain of human erythrocyte band 3. Science.

[123] Huang Y. (2003). Structure and mechanism of the glycerol-3-phosphate transporter from escherichia coli. Science.

[124] Deng D., Xu C., Sun P., Wu J., Yan C., Hu M., Yan N. (2014). Crystal structure of the human glucose transporter GLUT1. Nature.

[125] Avvaru B.S., Kim C.U., Sippel K.H., Gruner S.M., Agbandje-McKenna M., Silverman D.N., McKenna R. (2010). A short, Strong hydrogen bond in the active site of human carbonic anhydrase II. Biochemistry.

[126] Michalczyk R., Unkefer C.J., Bacik J.-P., Schrader T.E., Ostermann A., Kovalevsky A.Y., McKenna R., Fisher S.Z. (2015). Joint neutron crystallographic and NMR solution studies of Tyr residue ionization and hydrogen bonding: Implications for enzyme-mediated proton transfer. Proc. Natl. Acad. Sci. USA.

[127] Chiang W.L., Chu S.C., Lai J.C., Yang S.F., Chiou H.L., Hsieh Y.S. (2001). Alternations in quantities and activities of erythrocyte cytosolic carbonic anhydrase isoenzymes in glucose-6-phosphate dehydrogenase-deficient individuals. Clin. Chim. Acta Int. J. Clin. Chem..

[128] Maren T.H., Swenson E.R. (1980). A comparative study of the kinetics of the Bohr effect in vertebrates. J. Physiol..

[129] Swenson E.R. (2000). Respiratory and renal roles of carbonic anhydrase in gas exchange and acid-base regulation. The Carbonic Anhydrases.

[130] Brown B.F., Quon A., Dyck J.R.B., Casey J.R. (2012). Carbonic anhydrase II promotes cardiomyocyte hypertrophy. Can. J. Physiol. Pharmacol..

[131] Kuo W.-H., Yang S.-F., Hsieh Y.-S., Tsai C.-S., Hwang W.-L., Chu S.-C. (2005). Differential expression of carbonic anhydrase isoenzymes in various types of anemia. Clin. Chim. Acta Int. J. Clin. Chem..

[132] Gilmour K.M. (2010). Perspectives on carbonic anhydrase. Comp. Biochem. Physiol. A Mol. Integr. Physiol..

[133] Becker H.M., Deitmer J.W. (2007). Carbonic anhydrase II increases the activity of the human electrogenic Na^+^/HCO_3_^−^ cotransporter. J. Biol. Chem..

[134] Becker H.M., Deitmer J.W. (2008). Nonenzymatic proton handling by carbonic anhydrase II during H^+^-lactate cotransport via monocarboxylate transporter 1. J. Biol. Chem..

[135] Stridh M.H., Alt M.D., Wittmann S., Heidtmann H., Aggarwal M., Riederer B., Seidler U., Wennemuth G., McKenna R., Deitmer J.W. (2012). Lactate flux in astrocytes is enhanced by a non-catalytic action of carbonic anhydrase II: CAII enhances lactate transport in astrocytes. J. Physiol..

[136] Uhlén M., Björling E., Agaton C., Szigyarto C.A.-K., Amini B., Andersen E., Andersson A.-C., Angelidou P., Asplund A., Asplund C. (2005). A human protein atlas for normal and cancer tissues based on antibody proteomics. Mol. Cell. Proteom..

[137] Uhlen M., Oksvold P., Fagerberg L., Lundberg E., Jonasson K., Forsberg M., Zwahlen M., Kampf C., Wester K., Hober S. (2010). Towards a knowledge-based human protein atlas. Nat. Biotechnol..

[138] Uhlen M., Fagerberg L., Hallstrom B.M., Lindskog C., Oksvold P., Mardinoglu A., Sivertsson A., Kampf C., Sjostedt E., Asplund A. (2015). Tissue-based map of the human proteome. Science.

[139] Uhlen M., Zhang C., Lee S., Sjöstedt E., Fagerberg L., Bidkhori G., Benfeitas R., Arif M., Liu Z., Edfors F. (2017). A pathology atlas of the human cancer transcriptome. Science.

[140] Thul P.J., Åkesson L., Wiking M., Mahdessian D., Geladaki A., Ait Blal H., Alm T., Asplund A., Björk L., Breckels L.M. (2017). A subcellular map of the human proteome. Science.

[141] Cerami E., Gao J., Dogrusoz U., Gross B.E., Sumer S.O., Aksoy B.A., Jacobsen A., Byrne C.J., Heuer M.L., Larsson E. (2012). The cBio Cancer Genomics Portal: An open platform for exploring multidimensional cancer genomics data: Figure 1. Cancer Discov..

[142] Gao J., Aksoy B.A., Dogrusoz U., Dresdner G., Gross B., Sumer S.O., Sun Y., Jacobsen A., Sinha R., Larsson E. (2013). Integrative Analysis of Complex Cancer Genomics and Clinical Profiles Using the cBioPortal. Sci. Signal..

[143] Zheng Y., Xu B., Zhao Y., Gu H., Li C., Wang Y., Chang X. (2015). CA1 contributes to microcalcification and tumourigenesis in breast cancer. BMC Cancer.

[144] Wang D., Lu X., Zhang X., Li Z., Li C. (2016). Carbonic anhydrase 1 is a promising biomarker for early detection of non-small cell lung cancer. Tumor Biol..

[145] Takakura M., Yokomizo A., Tanaka Y., Kobayashi M., Jung G., Banno M., Sakuma T., Imada K., Oda Y., Kamita M. (2012). Carbonic anhydrase I as a new plasma biomarker for prostate cancer. ISRN Oncol..

[146] Zhou R., Huang W., Yao Y., Wang Y., Li Z., Shao B., Zhong J., Tang M., Liang S., Zhao X. (2013). CA II, a potential biomarker by proteomic analysis, exerts significant inhibitory effect on the growth of colorectal cancer cells. Int. J. Oncol..

[147] Parkkila S., Lasota J., Fletcher J.A., Ou W., Kivelä A.J., Nuorva K., Parkkila A.-K., Ollikainen J., Sly W.S., Waheed A. (2010). Carbonic anhydrase II. A novel biomarker for gastrointestinal stromal tumors. Mod. Pathol..

[148] Järvinen P., Kivelä A.J., Nummela P., Lepistö A., Ristimäki A., Parkkila S. (2017). Carbonic anhydrase II: A novel biomarker for pseudomyxoma peritonei. APMIS.

[149] Waterman E.A., Cross N.A., Lippitt J.M., Cross S.S., Rehman I., Holen I., Hamdy F.C., Eaton C.L. (2007). The antibody MAB8051 directed against osteoprotegerin detects carbonic anhydrase II: Implications for association studies with human cancers. Int. J. Cancer.

[150] Liu L.-C. (2013). Overexpression of carbonic anhydrase II and Ki-67 proteins in prognosis of gastrointestinal stromal tumors. World J. Gastroenterol..

[151] Liu C.-M., Lin Y.-M., Yeh K.-T., Chen M.-K., Chang J.-H., Chen C.-J., Chou M.-Y., Yang S.-F., Chien M.-H. (2012). Expression of carbonic anhydrases I/II and the correlation to clinical aspects of oral squamous cell carcinoma analyzed using tissue microarray: CA I/II and correlation to OSCC. J. Oral Pathol. Med..

[152] Harju A.-K., Bootorabi F., Kuuslahti M., Supuran C.T., Parkkila S. (2013). Carbonic anhydrase III: A neglected isozyme is stepping into the limelight. J. Enzyme Inhib. Med. Chem..

[153] Shi C., Uda Y., Dedic C., Azab E., Sun N., Hussein A.I., Petty C.A., Fulzele K., Mitterberger-Vogt M.C., Zwerschke W. (2018). Carbonic anhydrase III protects osteocytes from oxidative stress. FASEB J..

[154] Dai H.-Y., Hong C.-C., Liang S.-C., Yan M.-D., Lai G.-M., Cheng A.-L., Chuang S.-E. (2008). Carbonic anhydrase III promotes transformation and invasion capability in hepatoma cells through FAK signaling pathway. Mol. Carcinog..

[155] Bootorabi F., Haapasalo J., Smith E., Haapasalo H., Parkkila S. (2011). Carbonic anhydrase VII—A potential prognostic marker in gliomas. Health.

[156] Hilvo M., Innocenti A., Monti S.M., De Simone G., Supuran C.T., Parkkila S. (2008). Recent advances in research on the most novel carbonic anhydrases, CA XIII and XV. Curr. Pharm. Des..

[157] Shah G.N., Hewett-Emmett D., Grubb J.H., Migas M.C., Fleming R.E., Waheed A., Sly W.S. (2000). Mitochondrial carbonic anhydrase CA VB: Differences in tissue distribution and pattern of evolution from those of CA VA suggest distinct physiological roles. Proc. Natl. Acad. Sci. USA.

[158] Nishimori I., Vullo D., Innocenti A., Scozzafava A., Mastrolorenzo A., Supuran C.T. (2005). Carbonic Anhydrase Inhibitors. The Mitochondrial Isozyme VB as a new target for sulfonamide and sulfamate inhibitors. J. Med. Chem..

[159] Arechederra R.L., Waheed A., Sly W.S., Supuran C.T., Minteer S.D. (2013). Effect of sulfonamides as carbonic anhydrase VA and VB inhibitors on mitochondrial metabolic energy conversion. Bioorg. Med. Chem..

[160] Shah G.N., Rubbelke T.S., Hendin J., Nguyen H., Waheed A., Shoemaker J.D., Sly W.S. (2013). Targeted mutagenesis of mitochondrial carbonic anhydrases VA and VB implicates both enzymes in ammonia detoxification and glucose metabolism. Proc. Natl. Acad. Sci. USA.

[161] Poulsen S.-A., Wilkinson B.L., Innocenti A., Vullo D., Supuran C.T. (2008). Inhibition of human mitochondrial carbonic anhydrases VA and VB with para-(4-phenyltriazole-1-yl)-benzenesulfonamide derivatives. Bioorg. Med. Chem. Lett..

[162] Lusty C.J. (1978). Carbamoylphosphate synthetase I of rat-liver mitochondria. Purification, properties, and polypeptide molecular weight. Eur. J. Biochem..

[163] Dodgson S.J., Forster R.E., Storey B.T. (1984). The role of carbonic anhydrase in hepatocyte metabolism. Ann. N. Y. Acad. Sci..

[164] Dodgson S.J., Forster R.E. (1986). Inhibition of CA V decreases glucose synthesis from pyruvate. Arch. Biochem. Biophys..

[165] Dodgson S.J. (1987). Inhibition of mitochondrial carbonic anhydrase and ureagenesis: A discrepancy examined. J. Appl. Physiol..

[166] Hazen S.A., Waheed A., Sly W.S., LaNoue K.F., Lynch C.J. (1996). Differentiation-dependent expression of CA V and the role of carbonic anhydrase isozymes in pyruvate carboxylation in adipocytes. FASEB J..

[167] Henry R.P. (1996). Multiple roles of carbonic anhydrase in cellular transport and metabolism. Annu. Rev. Physiol..

[168] Van Karnebeek C.D., Sly W.S., Ross C.J., Salvarinova R., Yaplito-Lee J., Santra S., Shyr C., Horvath G.A., Eydoux P., Lehman A.M. (2014). Mitochondrial Carbonic Anhydrase VA Deficiency Resulting from CA5A Alterations presents with hyperammonemia in early childhood. Am. J. Hum. Genet..

[169] Kivelä J., Parkkila S., Parkkila A.-K., Leinonen J., Rajaniemi H. (1999). Salivary carbonic anhydrase isoenzyme VI. J. Physiol..

[170] Frasseto F., Parisotto T.M., Peres R.C.R., Marques M.R., Line S.R.P., Nobre Dos Santos M. (2012). Relationship among salivary carbonic anhydrase VI activity and flow rate, biofilm pH and caries in primary dentition. Caries Res..

[171] Kimoto M., Kishino M., Yura Y., Ogawa Y. (2006). A role of salivary carbonic anhydrase VI in dental plaque. Arch. Oral Biol..

[172] Feeney E.L., Hayes J.E. (2014). Exploring associations between taste perception, oral anatomy and polymorphisms in the carbonic anhydrase (gustin) gene *CA6*. Physiol. Behav..

[173] Thatcher B.J., Doherty A.E., Orvisky E., Martin B.M., Henkin R.I. (1998). Gustin from human parotid saliva is carbonic anhydrase VI. Biochem. Biophys. Res. Commun..

[174] Karhumaa P., Leinonen J., Parkkila S., Kaunisto K., Tapanainen J., Rajaniemi H. (2001). The identification of secreted carbonic anhydrase VI as a constitutive glycoprotein of human and rat milk. Proc. Natl. Acad. Sci. USA.

[175] Ogawa Y., Matsumoto K., Maeda T., Tamai R., Suzuki T., Sasano H., Fernley R.T. (2002). Characterization of lacrimal gland carbonic anhydrase VI. J. Histochem. Cytochem..

[176] Leinonen J.S., Saari K.A., Seppänen J.M., Myllylä H.M., Rajaniemi H.J. (2004). Immunohistochemical demonstration of carbonic anhydrase isoenzyme VI (CA VI) expression in rat lower airways and lung. J. Histochem. Cytochem..

[177] Kivelä J., Parkkila S., Waheed A., Parkkila A.K., Sly W.S., Rajaniemi H. (1997). Secretory carbonic anhydrase isoenzyme (CA VI) in human serum. Clin. Chem..

[178] Li Z.-Q., Hu X.-P., Zhou J.-Y., Xie X.-D., Zhang J.-M. (2015). Genetic polymorphisms in the carbonic anhydrase VI gene and dental caries susceptibility. Genet. Mol. Res..

[179] Sengul F., Kilic M., Gurbuz T., Tasdemir S. (2016). Carbonic anhydrase VI gene polymorphism rs2274327 relationship between salivary parameters and dental-oral health status in children. Biochem. Genet..

[180] Dowd F.J. (1999). Saliva and dental caries. Dent. Clin. N. Am..

[181] Shatzman A.R., Henkin R.I. (1981). Gustin concentration changes relative to salivary zinc and taste in humans. Proc. Natl. Acad. Sci. USA.

[182] Henkin R.I., Lippoldt R.E., Bilstad J., Edelhoch H. (1975). A zinc protein isolated from human parotid saliva. Proc. Natl. Acad. Sci. USA.

[183] Innocenti A., Pastorekova S., Pastorek J., Scozzafava A., Simone G.D., Supuran C.T. (2009). The proteoglycan region of the tumor-associated carbonic anhydrase isoform IX acts as anintrinsic buffer optimizing CO_2_ hydration at acidic pH values characteristic of solid tumors. Bioorg. Med. Chem. Lett..

[184] Barker H., Aaltonen M., Pan P., Vähätupa M., Kaipiainen P., May U., Prince S., Uusitalo-Järvinen H., Waheed A., Pastoreková S. (2017). Role of carbonic anhydrases in skin wound healing. Exp. Mol. Med..

[185] Fujikawa-Adachi K., Nishimori I., Sakamoto S., Morita M., Onishi S., Yonezawa S., Hollingsworth M.A. (1999). Identification of carbonic anhydrase IV and VI mRNA expression in human pancreas and salivary glands. Pancreas.

[186] Wistrand P.J., Carter N.D., Conroy C.W., Mahieu I. (1999). Carbonic anhydrase IV activity is localized on the exterior surface of human erythrocytes. Acta Physiol. Scand..

[187] Sender S., Decker B., Fenske C.D., Sly W.S., Carter N.D., Gros G. (1998). Localization of carbonic anhydrase IV in rat and human heart muscle. J. Histochem. Cytochem..

[188] Parkkila S., Parkkila A.K., Juvonen T., Waheed A., Sly W.S., Saarnio J., Kaunisto K., Kellokumpu S., Rajaniemi H. (1996). Membrane-bound carbonic anhydrase IV is expressed in the luminal plasma membrane of the human gallbladder epithelium. Hepatology.

[189] Sender S., Gros G., Waheed A., Hageman G.S., Sly W.S. (1994). Immunohistochemical localization of carbonic anhydrase IV in capillaries of rat and human skeletal muscle. J. Histochem. Cytochem..

[190] Fleming R.E., Parkkila S., Parkkila A.K., Rajaniemi H., Waheed A., Sly W.S. (1995). Carbonic anhydrase IV expression in rat and human gastrointestinal tract regional, cellular, and subcellular localization. J. Clin. Investig..

[191] Purkerson J.M., Schwartz G.J. (2007). The role of carbonic anhydrases in renal physiology. Kidney Int..

[192] Alvarez B.V., Loiselle F.B., Supuran C.T., Schwartz G.J., Casey J.R. (2003). Direct extracellular interaction between carbonic anhydrase IV and the human NBC1 sodium/bicarbonate co-transporter. Biochemistry.

[193] Alvarez B.V., Vithana E.N., Yang Z., Koh A.H., Yeung K., Yong V., Shandro H.J., Chen Y., Kolatkar P., Palasingam P. (2007). Identification and characterization of a novel mutation in the carbonic anhydrase IV gene that causes retinitis pigmentosa. Invest. Ophthalmol. Vis. Sci..

[194] Yang Z., Alvarez B.V., Chakarova C., Jiang L., Karan G., Frederick J.M., Zhao Y., Sauvé Y., Li X., Zrenner E. (2005). Mutant carbonic anhydrase 4 impairs pH regulation and causes retinal photoreceptor degeneration. Hum. Mol. Genet..

[195] Parkkila S., Parkkila A.K., Rajaniemi H., Shah G.N., Grubb J.H., Waheed A., Sly W.S. (2001). Expression of membrane-associated carbonic anhydrase XIV on neurons and axons in mouse and human brain. Proc. Natl. Acad. Sci. USA.

[196] Kaunisto K., Parkkila S., Rajaniemi H., Waheed A., Grubb J., Sly W.S. (2002). Carbonic anhydrase XIV: Luminal expression suggests key role in renal acidification. Kidney Int..

[197] Juel C., Lundby C., Sander M., Calbet J.A.L., van Hall G. (2003). Human skeletal muscle and erythrocyte proteins involved in acid-base homeostasis: Adaptations to chronic hypoxia. J. Physiol..

[198] Vargas L.A., Alvarez B.V. (2012). Carbonic anhydrase XIV in the normal and hypertrophic myocardium. J. Mol. Cell. Cardiol..

[199] Pastorek J., Pastoreková S., Callebaut I., Mornon J.P., Zelník V., Opavský R., Zat’ovicová M., Liao S., Portetelle D., Stanbridge E.J. (1994). Cloning and characterization of MN, a human tumor-associated protein with a domain homologous to carbonic anhydrase and a putative helix-loop-helix DNA binding segment. Oncogene.

[200] Liao S.-Y., Lerman M.I., Stanbridge E.J. (2009). Expression of transmembrane carbonic anhydrases, CAIX and CAXII, in human development. BMC Dev. Biol..

[201] Ivanov S., Shu-Yuan L., Ivanova A., Danilkovitch-Miagkova A., Tarasova N., Weirich G., Merrill M.J., Proescholdt M.A., Oldfield E.H., Lee J. (2001). Expression of hypoxia inducible cell surface transmembrane carbonic anhydrases in human cancer. Am. J. Pathol..

[202] Radvak P., Repic M., Svastova E., Takacova M., Csaderova L., Strnad H., Pastorek J., Pastorekova S., Kopacek J. (2013). Suppression of carbonic anhydrase IX leads to aberrant focal adhesion and decreased invasion of tumor cells. Oncol. Rep..

[203] Csaderova L., Debreova M., Radvak P., Stano M., Vrestiakova M., Kopacek J., Pastorekova S., Svastova E. (2013). The effect of carbonic anhydrase IX on focal contacts during cell spreading and migration. Front. Physiol..

[204] Karhumaa P., Kaunisto K., Parkkila S., Waheed A., Pastoreková S., Pastorek J., Sly W.S., Rajaniemi H. (2001). Expression of the transmembrane carbonic anhydrases, CA IX and CA XII, in the human male excurrent ducts. Mol. Hum. Reprod..

[205] Parkkila S., Parkkila A.K., Saarnio J., Kivelä J., Karttunen T.J., Kaunisto K., Waheed A., Sly W.S., Türeci O., Virtanen I. (2000). Expression of the membrane-associated carbonic anhydrase isozyme XII in the human kidney and renal tumors. J. Histochem. Cytochem..

[206] Lee M., Vecchio-Pagán B., Sharma N., Waheed A., Li X., Raraigh K.S., Robbins S., Han S.T., Franca A.L., Pellicore M.J. (2016). Loss of carbonic anhydrase XII function in individuals with elevated sweat chloride concentration and pulmonary airway disease. Hum. Mol. Genet..

[207] Feldshtein M., Elkrinawi S., Yerushalmi B., Marcus B., Vullo D., Romi H., Ofir R., Landau D., Sivan S., Supuran C.T. (2010). Hyperchlorhidrosis caused by homozygous mutation in CA12, encoding carbonic anhydrase XII. Am. J. Hum. Genet..

[208] Capkova L., Koubkova L., Kodet R. (2014). Expression of carbonic anhydrase IX (CAIX) in malignant mesothelioma. An immunohistochemical and immunocytochemical study. Neoplasma.

[209] Yang J.-S., Chen M.-K., Yang S.-F., Chang Y.-C., Su S.-C., Chiou H.-L., Chien M.-H., Lin C.-W. (2014). Increased expression of carbonic anhydrase IX in oral submucous fibrosis and oral squamous cell carcinoma. Clin. Chem. Lab. Med..

[210] Jomrich G., Jesch B., Birner P., Schwameis K., Paireder M., Asari R., Schoppmann S.F. (2014). Stromal expression of carbonic anhydrase IX in esophageal cancer. Clin. Transl. Oncol..

[211] Wykoff C.C., Beasley N.J., Watson P.H., Turner K.J., Pastorek J., Sibtain A., Wilson G.D., Turley H., Talks K.L., Maxwell P.H. (2000). Hypoxia-inducible expression of tumor-associated carbonic anhydrases. Cancer Res..

[212] Kopacek J., Barathova M., Dequiedt F., Sepelakova J., Kettmann R., Pastorek J., Pastorekova S. (2005). MAPK pathway contributes to density- and hypoxia-induced expression of the tumor-associated carbonic anhydrase IX. Biochim. Biophys. Acta BBA—Gene Struct. Expr..

[213] Kaluz S., Kaluzová M., Chrastina A., Olive P.L., Pastoreková S., Pastorek J., Lerman M.I., Stanbridge E.J. (2002). Lowered oxygen tension induces expression of the hypoxia marker MN/carbonic anhydrase IX in the absence of hypoxia-inducible factor 1 alpha stabilization: A role for phosphatidylinositol 3′-kinase. Cancer Res..

[214] Swayampakula M., McDonald P.C., Vallejo M., Coyaud E., Chafe S.C., Westerback A., Venkateswaran G., Shankar J., Gao G., Laurent E.M.N. (2017). The interactome of metabolic enzyme carbonic anhydrase IX reveals novel roles in tumor cell migration and invadopodia/MMP14-mediated invasion. Oncogene.

[215] Ditte P., Dequiedt F., Svastova E., Hulikova A., Ohradanova-Repic A., Zatovicova M., Csaderova L., Kopacek J., Supuran C.T., Pastorekova S. (2011). Phosphorylation of carbonic anhydrase IX controls its ability to mediate extracellular acidification in hypoxic tumors. Cancer Res..

[216] Dorai T., Sawczuk I.S., Pastorek J., Wiernik P.H., Dutcher J.P. (2005). The role of carbonic anhydrase IX overexpression in kidney cancer. Eur. J. Cancer.

[217] Nasu K., Yamaguchi K., Takanashi T., Tamai K., Sato I., Ine S., Sasaki O., Satoh K., Tanaka N., Tanaka Y. (2017). Crucial role of carbonic anhydrase IX in tumorigenicity of xenotransplanted adult T-cell leukemia-derived cells. Cancer Sci..

[218] Li Y., Dong M., Sheng W., Huang L. (2016). Roles of carbonic anhydrase IX in development of pancreatic cancer. Pathol. Oncol. Res..

[219] Li G., Feng G., Zhao A., Péoc’h M., Cottier M., Mottet N. (2017). CA9 as a biomarker in preoperative biopsy of small solid renal masses for diagnosis of clear cell renal cell carcinoma. Biomarkers.

[220] Smith A.D., Truong M., Bristow R., Yip P., Milosevic M.F., Joshua A.M. (2016). The utility of serum CA9 for prognostication in prostate cancer. Anticancer Res..

[221] De Martino M., Lucca I., Mbeutcha A., Wiener H.G., Haitel A., Susani M., Shariat S.F., Klatte T. (2015). Carbonic anhydrase IX as a diagnostic urinary marker for urothelial bladder cancer. Eur. Urol..

[222] Huang W.-J., Jeng Y.-M., Lai H.-S., Fong I.-U., Sheu F.-Y. B., Lai P.-L., Yuan R.-H. (2015). Expression of hypoxic marker carbonic anhydrase IX predicts poor prognosis in resectable hepatocellular carcinoma. PLoS ONE.

[223] Ilie M.I., Hofman V., Ortholan C., Ammadi R.E., Bonnetaud C., Havet K., Venissac N., Mouroux J., Mazure N.M., Pouysségur J. (2011). Overexpression of carbonic anhydrase XII in tissues from resectable non-small cell lung cancers is a biomarker of good prognosis. Int. J. Cancer.

[224] Kobayashi M., Matsumoto T., Ryuge S., Yanagita K., Nagashio R., Kawakami Y., Goshima N., Jiang S.-X., Saegusa M., Iyoda A. (2012). CAXII is a sero-diagnostic marker for lung cancer. PLoS ONE.

[225] Yoo C.W., Nam B.-H., Kim J.-Y., Shin H.-J., Lim H., Lee S., Lee S.-K., Lim M.-C., Song Y.-J. (2010). Carbonic anhydrase XII expression is associated with histologic grade of cervical cancer and superior radiotherapy outcome. Radiat. Oncol. Lond. Engl..

[226] Chien M.-H., Ying T.-H., Hsieh Y.-H., Lin C.-H., Shih C.-H., Wei L.-H., Yang S.-F. (2012). Tumor-associated carbonic anhydrase XII is linked to the growth of primary oral squamous cell carcinoma and its poor prognosis. Oral Oncol..

[227] Kopecka J., Campia I., Jacobs A., Frei A.P., Ghigo D., Wollscheid B., Riganti C. (2015). Carbonic anhydrase XII is a new therapeutic target to overcome chemoresistance in cancer cells. Oncotarget.

